# Calpain 2 Isoform‐Specific Cleavage of Filamin A Enhances HIF1α Nuclear Translocation, Promoting Metastasis in Triple‐Negative Breast Cancer

**DOI:** 10.1002/mco2.70147

**Published:** 2025-03-27

**Authors:** Kyung‐Hwa Jeon, Seojeong Park, Eun Seon Pak, Jeong‐Ahn Kim, Yi Liu, Soo‐Yeon Hwang, Younghwa Na, Youngjoo Kwon

**Affiliations:** ^1^ College of Pharmacy Graduate School of Pharmaceutical Sciences Ewha Womans University Seoul Republic of Korea; ^2^ Gradutate Program in Innovative Biomaterials Convergence Ewha Womans University Seoul Republic of Korea; ^3^ College of Pharmacy CHA University Gyeongghi‐do Republic of Korea

**Keywords:** calpain 2, calpain inhibitor, epithelial–mesenchymal transition, metastasis, triple‐negative breast cancer

## Abstract

Triple‐negative breast cancer (TNBC) remains a challenge due to its aggressive nature and limited therapeutic options. Calpain 2, a member of the calcium‐dependent cysteine protease family, is particularly associated with poor prognosis in TNBC. This study explores the isoform‐specific role of calpain 2 in TNBC, examining its correlation with prognosis and its mechanistic impact on metastasis. Bioinformatic analyses, including Kaplan–Meier survival plots, univariate Cox proportional analysis, and gene set enrichment analysis (GSEA), assessed *CAPN2* expression and its association with mesenchymal genes in TNBC. Results of cell‐based experiments with *CAPN2* knockdown or overexpression indicate that elevated *CAPN2* expression correlates with poor clinical outcomes and enhanced metastatic potential in TNBC. *CAPN2* knockdown inhibited the epithelial–mesenchymal transition (EMT), reducing cancer cell proliferation, migration, and invasion. Calpain 2 downregulation reversed the EMT by reducing isoform‐specific cleavage of filamin A, HIF1α nuclear localization and *TWIST1* transcription. CNa **29**, a calpain 2‐specific inhibitor, reduced cancer cell proliferation, decreased filamin A cleavage, downregulated *TWIST1* expression, and significantly retarded metastasis,. In conclusion, calpain 2 plays a critical role in TNBC progression by modulating HIF1α and *TWIST1*, to promote the EMT and metastasis. Isoform‐selective inhibition of calpain 2 with CNa **29** presents a promising therapeutic strategy for managing TNBC.

## Graphical abstract

Calpain 2 cleaves Filamin A, leading to the activation of HIF‐1α, which translocates to the nucleus and interacts with the hypoxia response element (HRE) on the TWIST1 gene promoter. The promoted expression of TWIST1 results in epithelial–mesenchymal transition (EMT), stemness, and metastasis. Isoform selective inhibition of calpain 2 specifically halts this cascade, thereby reducing metastasis.

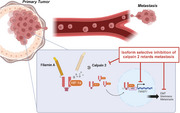

## 1 Introduction

Breast cancer is the most common type of cancer and the second leading cause of cancer‐related fatalities in women globally [1]. Mortality rates have declined since 1990 due to expanded screening methods and effective adjuvant therapies, such as targeted therapy and immunotherapy [2, 3, 4]. However, breast cancer still presents unmet challenges in therapy, particularly in specific subtypes like triple‐negative breast cancer (TNBC). TNBC accounts for approximately 10%–17% of all breast cancer cases, with higher incidence rates among younger patients and certain ethnic groups such as African American and Hispanic populations [5, 6]. TNBC is aggressive, progresses rapidly, and has limited therapeutic options, leading to lower overall survival (OS) rates and challenges such as recurrence and chemoresistance [6].

Metastasis is a major cause of cancer‐related death [7]. In particular, metastasis is a major determinant of survival in TNBC patients. Metastasis in TNBC is known to be driven largely by phenotypical changes such as the EMT and cancer stemness [8, 9]. EMT‐related signature genes are closely associated with TNBC metastasis and its clinical outcomes, and they are considered as potential prognostic biomarkers for TNBC [9]. Cancer stemness is also a key driver of tumor recurrence and progression, showing a strong link to drug resistance [8]. Various studies have focused on signaling pathways that regulate these major drivers in TNBC, including the JAK2‐STAT3 [10], PI3K/Akt/mTOR [11], and CDK1/USP29/TWIST1 [12].

Calpain, a family of calcium‐dependent cysteine proteases, includes more than 15 identified isoforms. Calpain 1 and 2 (also known as μ‐ and m‐calpain, respectively) are ubiquitously expressed and the most commonly studied [13]. Aberrant calpain expression is linked to various tumors, suggesting its potential involvement in tumorigenesis. Elevated calpain 1 levels are found in schwannomas, meningiomas, and renal carcinoma, while dysregulated calpain 2 is noted in colorectal adenocarcinomas, glioblastoma, prostate cancer, and renal cell carcinoma [14, 15, 16, 17]. Calpain 2 expression is associated with an unfavorable prognosis in basal‐like and TNBC [18]. Despite this association, the precise role and mechanisms of calpain 2 in TNBC are not fully understood.

In this study, we examined the role of calpain 2 and its correlation with the prognosis of mesenchymal TNBC. We explored how calpain 2 upregulation induces Twist expression, contributing to TNBC metastasis through filamin A cleavage and HIF1α nuclear localization. *CAPN2* knockdown or selective pharmacological inhibition of calpain 2 effectively suppressed TNBC metastasis driven by the HIF1α‐*TWIST1* axis.

## 2 Results

### 2.1 Elevated CAPN2 Expression Predicts Adverse Clinical Outcomes and Enhanced Metastatic Potential in TNBC

**FIGURE 1:**
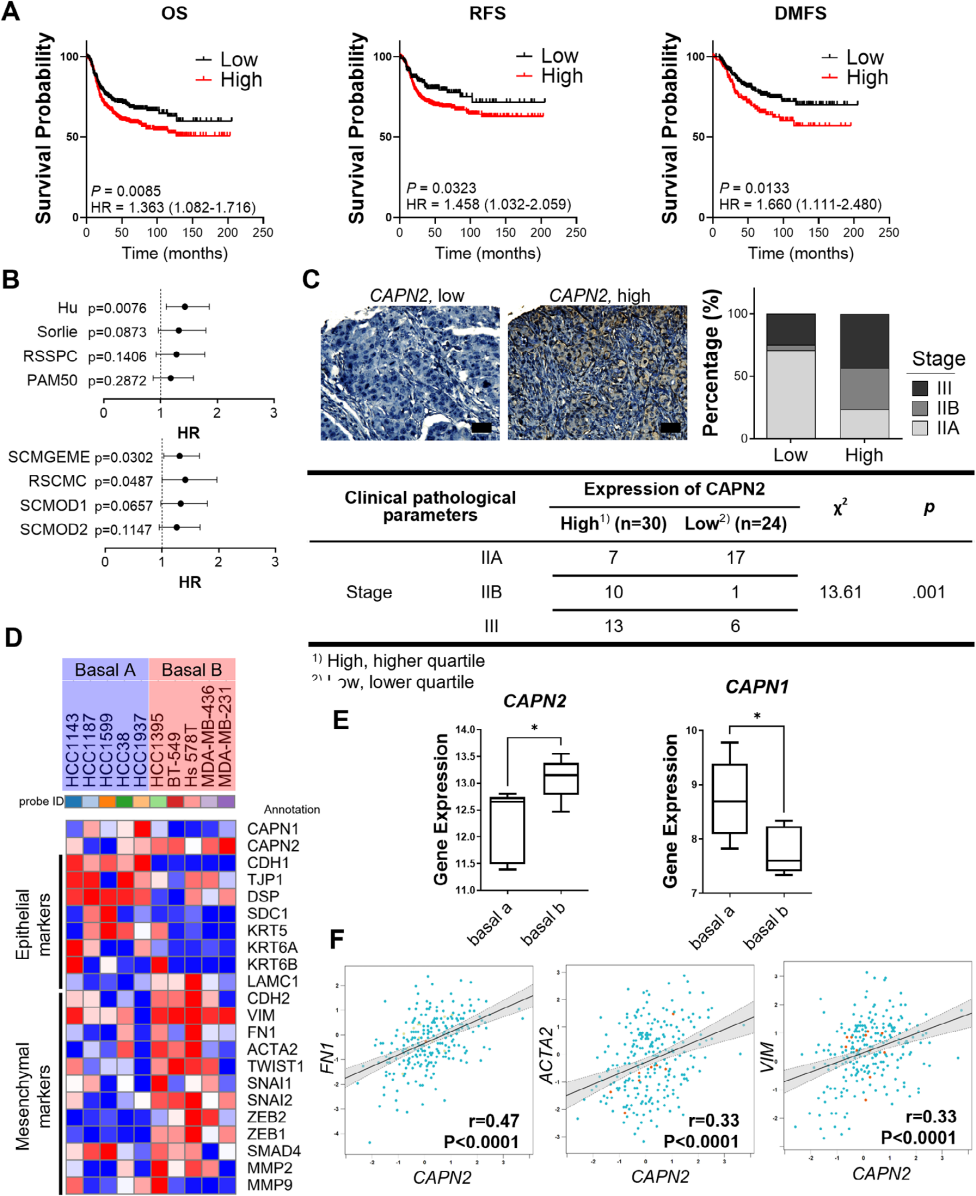
Prognostic significance of *CAPN2* expression and its correlation with mesenchymal phenotype in TNBC patients. (A) Kaplan–Meier analysis showing the correlation of *CAPN2* expression with overall, relapse‐free, and distant metastasis‐free survival. (B) Forest plot illustrating the results of *CAPN2* univariate Cox analysis by SSP molecular subtype. (C) Representative IHC images (scale bar = 50 µm) and correlation between calpain 2 expression and stage in tissue specimens of TNBC patients. Correlations between clinical pathological characteristics and expression of CAPN2 in TNBC tissues were analyzed using Pearson *χ* ^2^ test. (D) Heatmap displaying gene expressions related to EMT using data from the cancer cell line encyclopedia (CCLE). (E) Difference in calpain expression between the two subtypes of TNBC. (F) Correlation plot of *CAPN2* and mesenchymal genes, such as *FN1*, *ACTA2*, or *VIM* (bc‐ExGenMiner). *p < 0.05. The *p* values of unpaired two‐tailed Student's *t*‐test are shown (E).

Calpain 2 expression has been closely associated with an unfavorable prognosis in basal‐like and TNBC [18]. To substantiate this correlation, we conducted a database analysis prior to studying the underlying molecular mechanisms. Among basal‐like breast cancer patients with high gene expression of *CAPN2* (encoding calpain 2), there was a tendency toward lower OS, distant metastasis‐free survival (DMFS), and relapse‐free survival (RFS) (Figure 1A). In addition, the forest plot of *CAPN2* univariate Cox analysis by molecular subtype demonstrates that *CAPN2* expression increases the hazard ratio (HR) in basal‐like breast cancer (Figure 1B). Unlike in basal‐like breast cancer, *CAPN2* expression showed minimal impact on the risk of other subtypes of breast cancer, highlighting the potential significance of calpain 2 as a critical target only in TNBC (Figure S1A). In addition, upregulation of *CAPN1* (encoding calpain 1), another major isoform of calpain, did not affect any type of survival (Figure S1B), underscoring the isoform‐specific role of calpain 2 in influencing the cancer prognosis of TNBC. On the tissue microarray (TMA) slide containing TNBC biopsies, we observed a correlation between higher calpain 2 expression and tumor stage (Figure 1C). In TNBC patient tissues, elevated calpain 2 expression tended to align with higher tumor grades. Given that aggressiveness and metastasis are key factors in breast cancer prognosis [19], the increased risk and decreased survival may be associated with the mesenchymal phenotype. A heatmap analysis further revealed relationships between the expression of *CAPN2* and mesenchymal and epithelial markers (Figure 1D,E). Cell lines categorized as the mesenchymal subtype of TNBC, basal B type, showed a tendency toward higher levels of mesenchymal markers and lower levels of epithelial markers, accompanied by higher *CAPN2* and lower *CAPN1* expressions. Moreover, the expression of *CAPN2* in TNBC (IHC) and basal‐like (PAM50) tissues exhibited a positive correlation with mesenchymal genes, such as *FN1*, *ACTA2*, and *VIM* (Figures 1F and S2A). Notably, the correlation between *CAPN2* and mesenchymal genes in basal‐like breast cancer was more pronounced than in other subtypes of breast cancer (Figure S2B). These findings suggest that the expression of *CAPN2* but not *CAPN1* may potentially contribute to an increased risk of TNBC and basal‐like breast cancer characterized by enhanced mesenchymal gene expression.

### 2.2 *CAPN2* Downregulation Inhibited Cancer Cell Proliferation, Migration, Invasion, and the EMT

**FIGURE 2:**
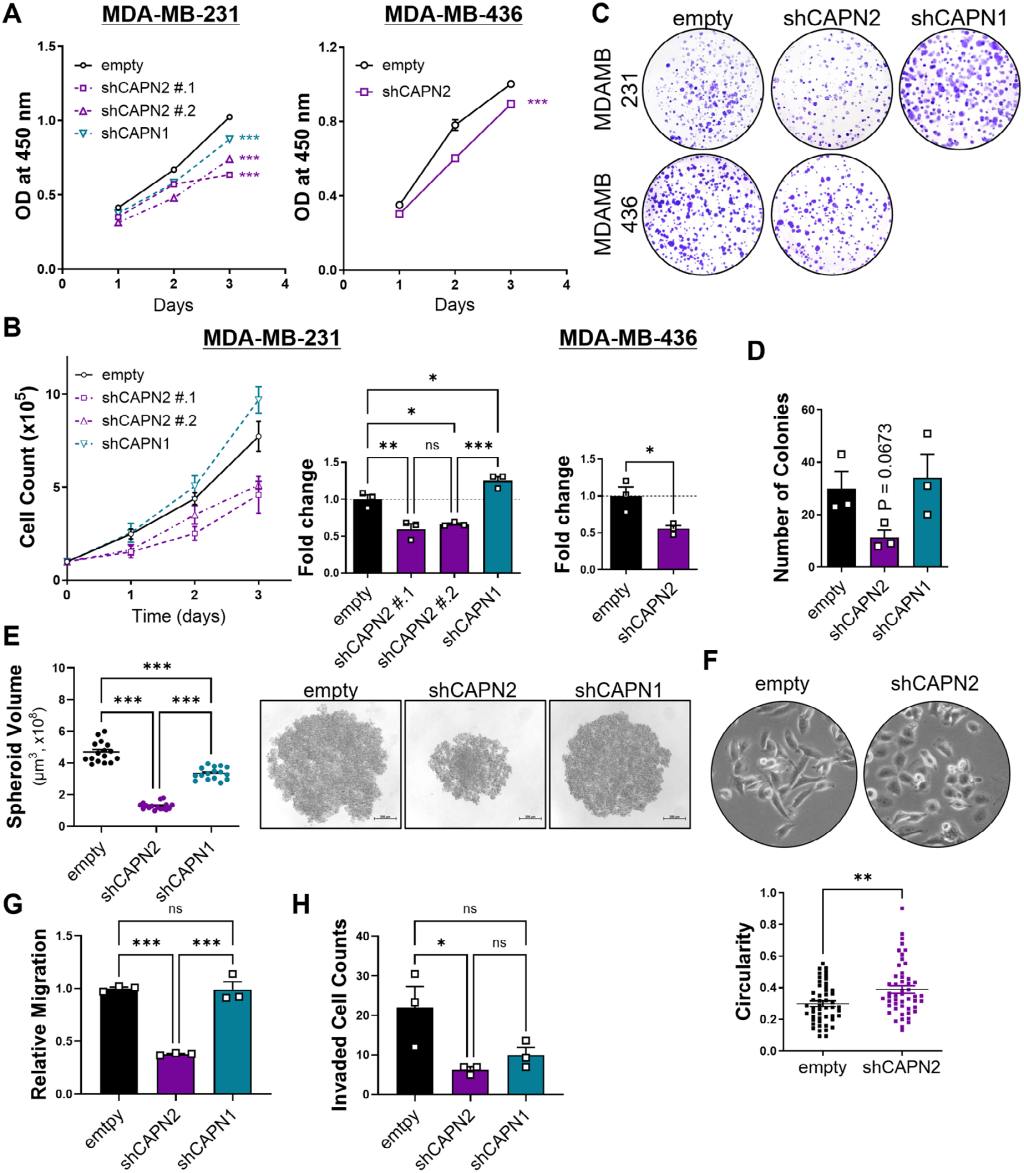
Essential role of *CAPN2* in the growth, migration, and invasion of TNBC cells. (A) WST assay of calpain stably knocked‐down and empty TNBC cells. (B) Evaluation of proliferation rate in calpain stably knocked‐down and empty TNBC cells. (C) Colony formation in TNBC cells with calpain downregulation. (D) Soft agar colony formation in calpain stably knocked‐down and control cells of MDA‐MB‐231. (E) Three‐dimensional cultured spheroidal growth of MDA‐MB‐231 cells with calpain knockdown. Representative images of spheroids in each group (scale bar = 200 µm). (F) Morphological alterations induced by *CAPN2* knockdown in MDA‐MB‐231 cells. Cell circularity of MDA‐MB‐231 cells was analyzed (*n* = 50). Representative phase images of each group of MDA‐MB‐231 cells grown in monolayer cultures are shown. (G) and (H) Transwell migration (G) and invasion (H) assay of MDA‐MB‐231 cells. *p* values of unpaired two‐tailed Student's *t*‐test (A‐right, B‐right, and F) and one‐way ANOVA (A‐left, B‐left, D, E, G, and H). *p < 0.05; **p < 0.01; ***p < 0.001; n.s., not significant. Data are presented as mean ± SEM.

**FIGURE 3:**
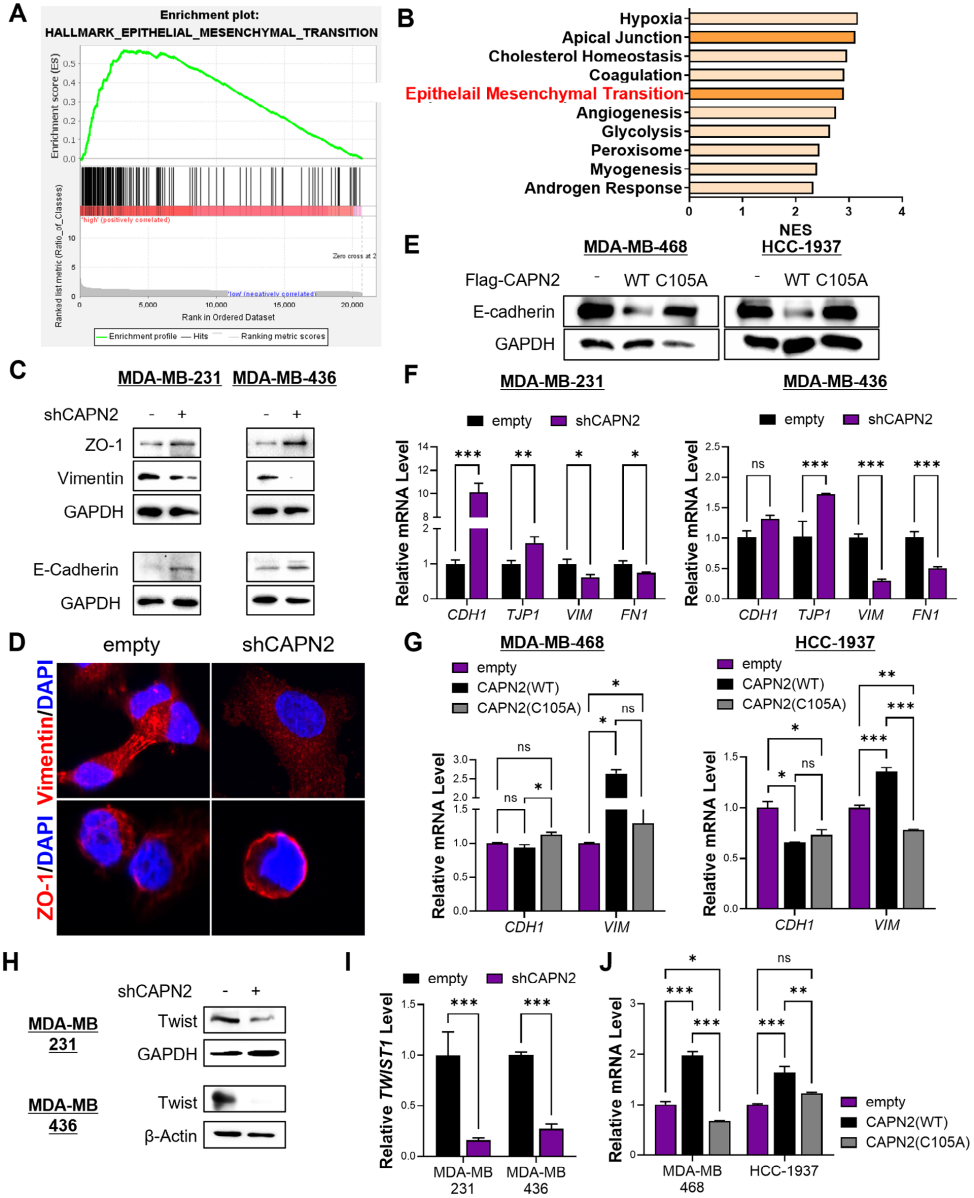
Reversal of EMT by *CAPN2* knockdown. (A) Enrichment of the EMT hallmark gene set observed in TNBC patients with high *CAPN2* expression. (B) Ranking of the top 10 highly enriched gene sets in highly *CAPN‐*expressing TNBC patients. (C) Confirmation of upregulation of epithelial markers and downregulation of mesenchymal markers in *CAPN2* knockdown cells. (D) Immunofluorescence analysis showing decreased vimentin and increased ZO‐1 expression in calpain 2‐downregulated MDA‐MB‐231 cells. (E) Evaluation of the epithelial marker, E‐cadherin, in calpain 2 overexpressing cells. (F) and (G) mRNA levels of epithelial and mesenchymal markers in TNBC cells with *CAPN2* knocked‐down (F) and ‐overexpressing d cells (G). (H) Downregulation in protein expression of Twist in *CAPN2* knocked‐down TNBC cells. (I) Downregulation in mRNA expression of *TWIST1* in *CAPN2* knocked‐down TNBC cells. (J) Upregulation of *TWIST1* in *CAPN2*‐overexpressed TNBC cell. The *p* values of unpaired two‐tailed Student's *t*‐test (F and H) and one‐way ANOVA (G and I). *p < 0.05; **p < 0.01; ***p < 0.001; n.s., not significant. Data are presented as mean ± SEM.

We aimed to elucidate the isoform‐specific roles of calpain 2 in the growth and metastasis of basal B or mesenchymal TNBC cells. Mesenchymal TNBC cell lines were selected and subjected to stable knockdown of *CAPN2* or *CAPN1* for further investigation (Figures 3 and S4). Notably, downregulating calpain 2, not calpain 1, significantly decreased both anchorage‐dependent (Figures 2A–C and S4A–C) and anchorage‐independent cellular growth (Figures 2D,E and S4D). Given that anchorage‐independent growth of cancer cells is strongly associated with invasiveness and metastasis [20], the isoform‐specific role of calpain 2 in cancer cell growth implies a potential connection between calpain 2 and the metastatic phenotype.

In addition to changes in cellular growth rate, cells with reduced calpain 2 expression exhibited morphological differences (Figure 2F). With *CAPN2* knockdown, cells displayed a cobblestone‐like morphology characterized by tight cell‐cell adhesion, while cells transduced with an empty vector retained their spindle‐like shape with a scattered distribution in culture. Calpain 2 downregulation significantly impaired not only cell migration (Figures 2G and S4E) but also invasion (Figure 2H), whereas calpain 1 downregulation showed little impact on these processes. These findings, along with the observed morphological differences and lesser growth upon *CAPN2* knockdown, support the previous clinical studies and database analysis suggesting that calpain 2 is closely related to cancer cell metastatic prognosis in TNBC, but calpain 1 is not.

Considering the observed effects on migration and invasion, we explored whether calpain 2 regulates the epithelial–mesenchymal transition (EMT). Gene set enrichment analysis (GSEA) of TNBC patients indicated the enrichment of EMT‐related gene sets in patients with high *CAPN2* levels (Figures 3A,B and S5A). Downregulation of calpain 2 resulted in reversal of the EMT, characterized by an increase in epithelial markers and a decrease in mesenchymal markers (Figures 3C,D and S5B). We also developed *CAPN2* overexpressing cellular models using basal A or epithelial TNBC cell lines and assessed the involvement of its enzymatic activity by overexpressing either wildtype *CAPN2* or a mutant form (C105A) (Figure S3). Overexpression of calpain 2 downregulated E‐cadherin expression, while an inactive mutant form of the enzyme did not decrease the E‐cadherin level (Figure 3E). These changes in EMT‐related protein markers were confirmed at the mRNA level (Figure 3F,G).

To identify the molecular mechanisms governing these changes induced by calpain 2 downregulation, we examined the levels of EMT‐related transcription factors. Among these factors, the protein level of Twist consistently showed reductions (Figures 3H and S5C). Twist is a pivotal transcription factor governing genes associated with EMT [21, 22, 23], and its protein levels are regulated not only by transcriptional control but also by modulation of stability via MAPK and AKT signaling pathways [24]. Although phosphorylated MAPK and AKT remained unaffected by calpain 2 downregulation, the mRNA level of *TWIST1* was significantly altered by *CAPN2* regulation (Figures S6A and 3I,J). Correspondingly, we found a positive correlation between *CAPN2* and *TWIST1* transcripts (Figure S7B). Moreover, gene signature analysis revealed that an elevated mean expression of the gene set consisting of *CAPN2* and *TWIST1* in metastatic TNBC tissues was notably more significant than the individual gene expressions (Figure S6C–E). Overall, these findings suggest that the expression of calpain 2 impacts the EMT by regulating Twist expression at the transcriptional level in mesenchymal TNBC cell lines.

### 2.3 *CAPN2* Downregulation Attenuates HIF1α Nuclear Localization and Limits *TWIST1* Expression in TNBC

**FIGURE 4:**
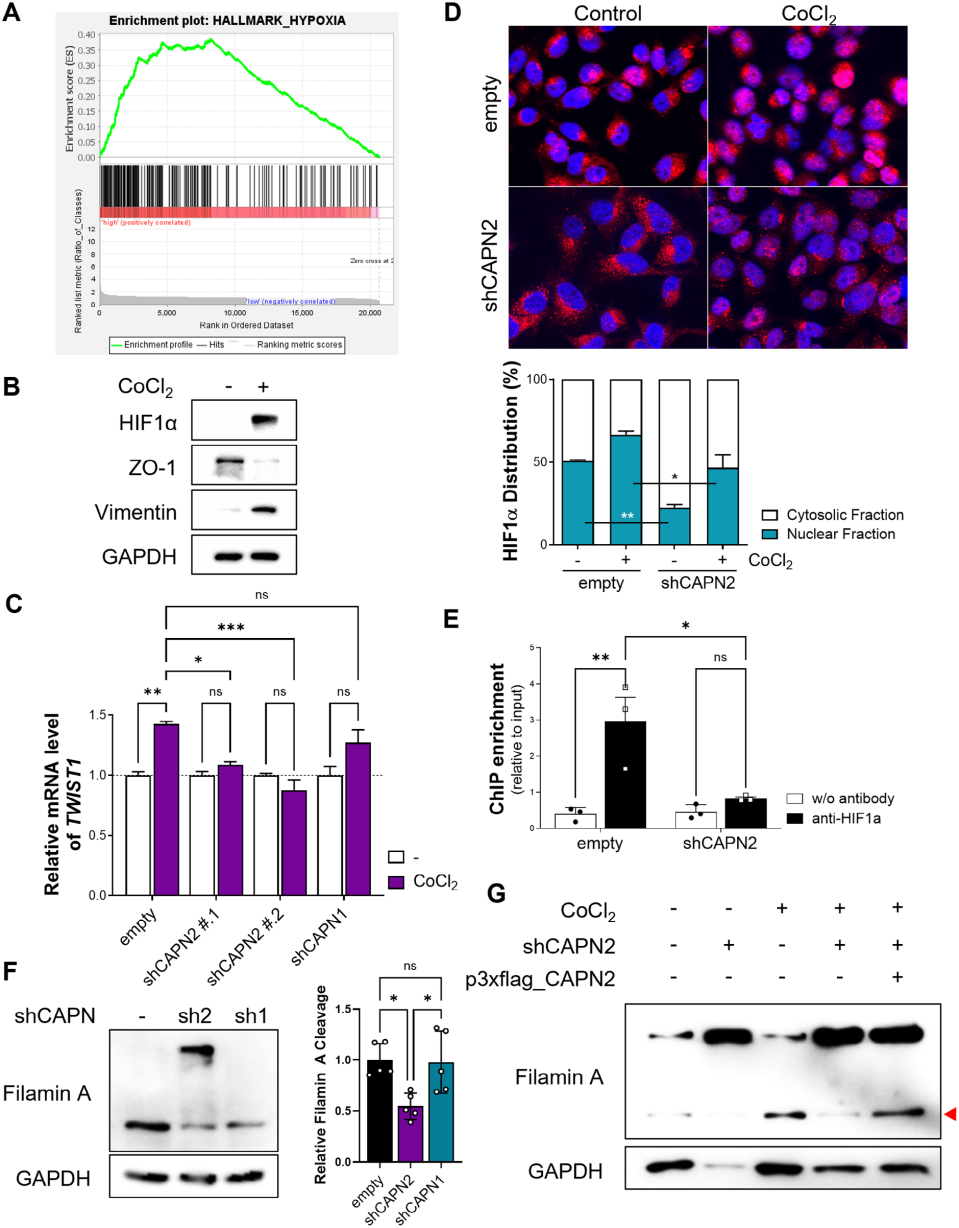
Calpain 2‐mediated HIF1α nuclear localization. (A) Enrichment of hypoxia‐related gene set observed in highly *CAPN2* expressing TNBC patients. (B) EMT induction by CoCl_2_ treatment was assessed. **(C)** Evaluation of nuclear localization of HIF1α in MDA‐MB‐231 cells through immunofluorescence experiment (scale bar = 20 µm). (D) Diminished cobalt chloride‐induced *TWIST1* elevation by *CAPN2* knockdown in MDA‐MB‐231 cells. (E) Quantification of HIF1α enrichment at the *TWIST1* promoter in MDA‐MB‐231 cells using chromatin immunoprecipitation (ChIP) assay. (F) Calpain isoform specific cleavage of filamin A in *CAPN1 and CAPN2* knockdown models of MDA‐MB‐231 cells. (G) Evaluation of calpain 2‐dependent cleavage of filamin A in MDA‐MB‐231 cells. The *p* values of two‐way ANOVA (C, D, and E). *p < 0.05; **p < 0.01; ***p < 0.001; n.s., not significant. Data are presented as mean ± SEM.

The observed decrease in *TWIST1* expression at the mRNA level (Figure 3I,J) prompted us to investigate potential transcriptional regulation of *TWIST1*. Through an analysis of the *TWIST1* promoter, we identified a set of candidate transcription factors (Table S3). Subsequently, we performed GSEA to uncover pathways associated with calpain 2 expression. In patients exhibiting elevated *CAPN2* levels, GSEA revealed a significant correlation with gene expression signatures characteristic of hypoxia (Figures 3, 4, and S5A). HIF1α, a well‐known transcription factor activated by hypoxic stimuli, regulates diverse gene expressions [25]. *TWIST1* is one of the target genes regulated by HIF1α through direct binding to the hypoxia‐response element (HRE) in the *TWIST1* promoter [26]. It is known that the functionality of HIF1α as a transcription factor is influenced by its protein stability and subcellular localization [27]. Prior to the evaluation of HIF1α involvement in the regulation of *TWIST1* expression, the role of HIF1α in EMT regulation was confirmed (Figure 4B). CoCl_2_ treatment, known to stabilize HIF1α and enhance its activity [28], induces EMT by downregulating epithelial markers and upregulating mesenchymal markers. We subsequently assessed the nuclear localization of HIF1α and its impact on *TWIST1* expression under CoCl_2_‐treated condition. Calpain 2 knockdown significantly reduced the nuclear proportion of HIF1α compared to control cells (Figures 4C and S7A). This reduction was further supported by RNA‐seq analysis, which demonstrated calpain 2‐dependent gene set enrichment in the hypoxia hallmark set (data not shown). CoCl_2_ treatment induced nuclear accumulation of HIF1α and upregulated *TWIST1* expression, which was attenuated by calpain 2 downregulation (Figure 4D). The chromatin immunoprecipitation (ChIP) assay revealed that calpain 2 knockdown resulted in the loss of HIF1α binding to the *TWIST1* promoter (Figure 4E). These results indicate that the reduction of Twist in calpain 2 knockdown cells is attributed to alteration in the subcellular localization of HIF1α.

Filamin A cleavage has been reported as a regulator of HIF1α subcellular localization [29]. Therefore, we sought to determine whether the C‐terminal fragment of filamin A contributes to the nuclear localization of HIF1α. In control and *CAPN1* knockdown cells, significant cleavage of filamin A was observed; this cleavage was markedly reduced in *CAPN2* knockdown cells (Figure 4F). Moreover, the elevation of the C‐terminal fragment of filamin A induced by CoCl_2_ treatment, mimicking a hypoxic condition, was attenuated by calpain 2 knockdown, and restored by reexpression of calpain 2 via transient transfection of a calpain 2 expression vector (Figures 4G, S7B,C). Our findings are consistent with previous studies demonstrating that the calpain‐induced production of C‐terminal fragments of filamin A promotes the nuclear accumulation of HIF1α. In addition, inhibition of filamin A cleavage by a calpain protease inhibitor supports this mechanism, although the isotype specificity of calpain was not specified [29]. Considering that HIF1α activation occurs under hypoxic conditions commonly observed in solid tumors [30], we examined hypoxic conditions within three‐dimensional tumor spheroids to simulate the hypoxic microenvironment of tumoral tissue (Figure S8). Our analysis revealed a hypoxic inner region characterized by nuclear HIF1α localization in three‐dimensional cultured tumor spheroids, which was notably decreased in *CAPN2* knockdown tumor spheroids. Collectively, the downregulation of *CAPN2* resulted in reduced filamin A cleavage, hindering the nuclear translocation and activity of HIF1α and leading to a decrease in *TWIST1* expression and inhibition of the EMT process.

### 2.4 *CAPN2* Knockdown Hinders Cancer Stemness and Reduces Metastatic Potential in TNBC

Tumor spheroids present both mesenchymal traits and stem cell‐like properties. Cancer stemness is associated with a higher metastatic incidence, drug resistance, and poor prognosis in cancer treatment [31]. To assess the relationship between *CAPN2* and cancer cell stemness, we analyzed the gene expressions of primary tumors and tumor spheroids. Compared to primary tumors, tumor spheroids showed increased levels of mesenchymal and HIF1α target genes, along with elevated *CAPN2* expression (Figure S9A,B). In breast cancer, tumor spheroids are called mammospheres, and their growth is indicative of self‐renewal capacity and cancer stemness [32]. In a mammosphere assay, the size of mammospheres was not increased in *CAPN2* knockdown cells, while it significantly increased in control and *CAPN1* knockdown cells (Figure S9C,D). Furthermore, cancer stemness markers, such as Oct4, Nanog, and Sox2, were downregulated in *CAPN2* knockdown cells (Figure S9E,F). Notably, *CAPN2* downregulation resulted in increased drug sensitivity (Figure S9G).

**FIGURE 5:**
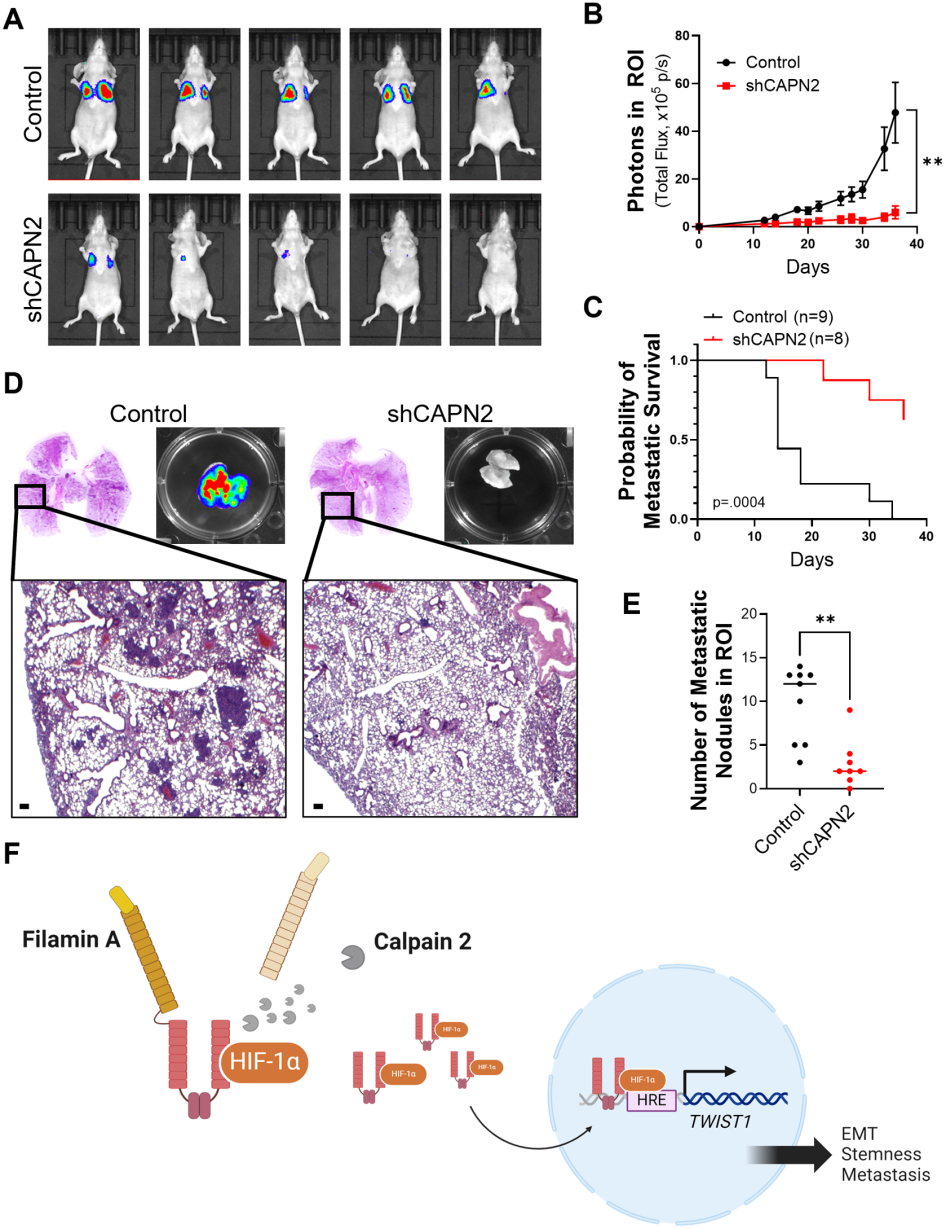
Attenuation of lung metastasis by calpain 2 inhibition in an in vivo metastasis model. (A) Bioluminescent images showing metastatic tumors in different groups. (B) Quantification of bioluminescent signals to analyze metastatic tumors. (C) Metastatic survival analysis of mouse model conducted in the two groups. (D) Representative H&E images displaying metastatic lesions in lung tissues. (E) Quantification of metastatic nodules in lung tissues. (F) Schematic illustration depicting the involvement of calpain 2 in TNBC metastasis. The *p* values of unpaired two‐tailed Student's *t*‐test (B, C, and D). **p < 0.01. Data are presented as mean ± SEM.

Next, we conducted an in vivo metastatic assay to determine whether the observed reduction in metastatic molecular phenotypes affected the metastasis in the in vivo model (Figures 5 and S10). A significant reduction in pulmonary metastasis was observed upon *CAPN2* knockdown. The metastatic incidence was notably retarded in the *CAPN2* knockdown group compared to the control group (Figure 5A–E). The mechanisms underlying the reduction in cancer stemness and metastatic potential by *CAPN2* knockdown are briefly summarized in Figure 5F.

### 2.5 Isoform‐Selective Pharmacological Inhibition of Calpain 2 Retards Metastasis in an In Vivo TNBC Metastasis Model

**FIGURE 6:**
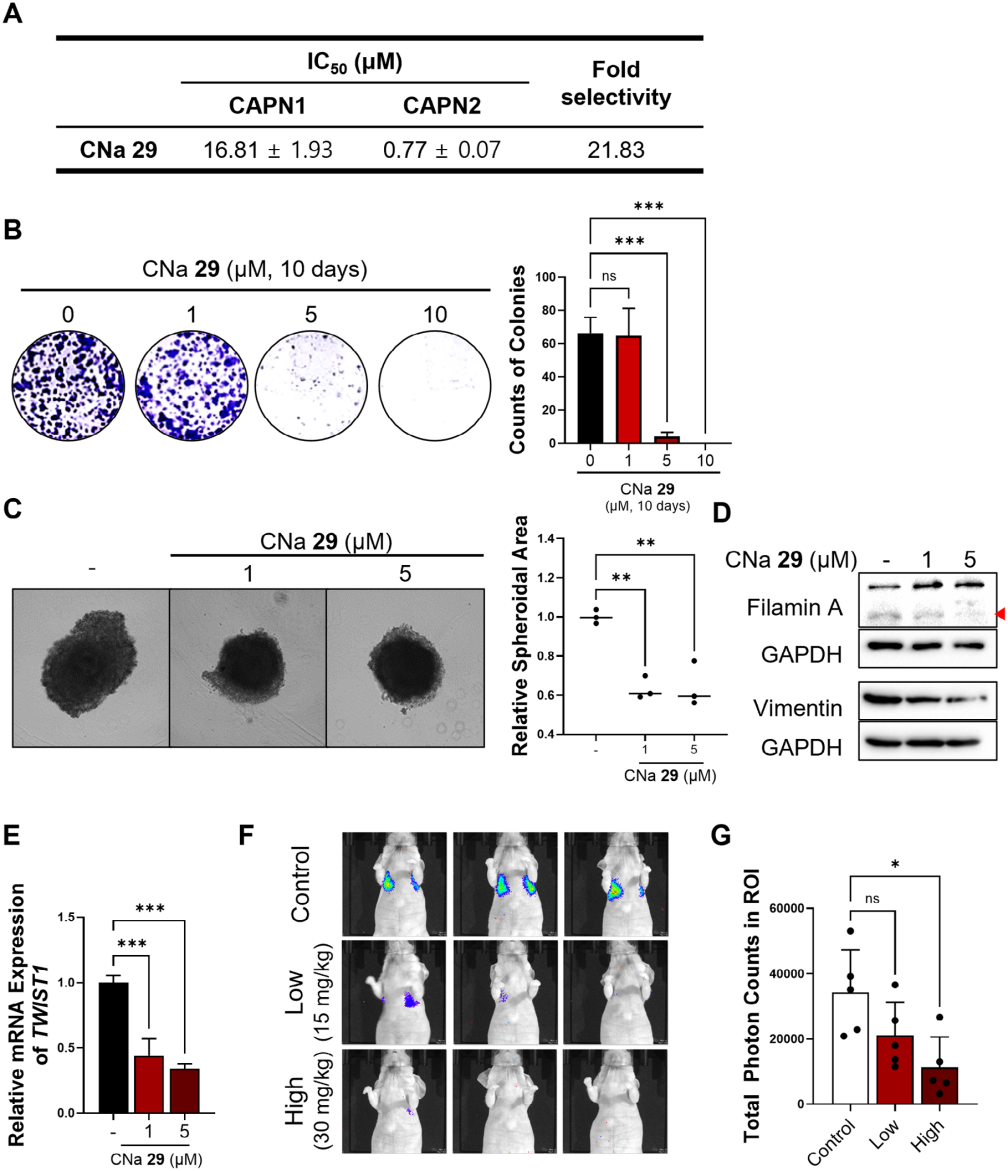
Inhibitory activity of CNa **29** on calpain activity, cell growth, and metastasis. (A) Calpain isoform selective inhibitory activity of CNa **29**. (B) and (C) Evaluation of cellular growth inhibitory activity of CNa **29** using clonogenic assay (B) and 3D spheroids (C). (D) Western blot analysis of MDA‐MB‐231 cells treated with CNa **29**. Inhibition of filamin A cleavage and downregulation of vimentin protein level were evaluated. (E) Assessment of *TWIST1* transcription after treatment of CNa **29**. Decrease in mRNA levels of *TWIST1* by CNa **29** treatment was observed. (F) and (G) Representative bioluminescence images (F) and normalized bioluminescence signal (G) for lung metastases after tail vein injection of MDA‐MB‐231‐luc cells, followed by treatment of intraperitoneal (i.p.) injection with CNa **29**. The *p* values of one way ANOVA (B, C, E, and G). *p < 0.05; **p < 0.01; ***p < 0.001; n.s., not significant. Data are presented as mean ± SEM.

The results in this study strongly indicate that calpain‐mediated filamin A cleavage and HIF1α activation, leading to tumor metastasis, are primarily regulated by calpain 2, rather than calpain 1 (Figure 5F). Given this isoform‐specific function of calpain 2 in promoting TNBC metastasis, employing isoform‐selective calpain 2 inhibitors could offer a precise and effective therapeutic approach for managing the metastatic progression of TNBC. In our earlier research, various calpain inhibitors were explored [33, 34, 35, 36, 37, 38]. Among them, CNa **29** [39] exhibited significant specificity toward calpain 2, displaying greater than 20‐fold stronger activity compared to calpain 1 (Figure 6A). To assess the impact of isoform‐selective pharmacological inhibition of calpain 2 on tumor metastasis, we evaluated the effects of CNa **29** in TNBC cells. Without altering the protein level of calpain 2 (Figure S11A), CNa **29** retarded cancer cell growth in both 2D and 3D culture systems (Figure 6B,C). MDA‐MB‐231 cells were treated with CNa **29** to determine whether pharmacological inhibition of calpain 2 by CNa **29** prevents filamin A cleavage‐induced HIF1α nuclear translocation, *TWIST1* expression, EMT, cancer stemness, and metastasis. CNa **29** significantly reduced filamin A cleavage, vimentin expression, *TWIST1* transcription, and the expressions of genes related to cancer stemness and increased the efficacy of doxorubicin, a chemotherapy drug commonly used for TNBC in clinical settings (Figures 6D,E and S11B–D). Furthermore, CNa **29** exhibited a significant reduction in lung colonization in an in vivo metastasis model (Figure 6F,G) without loss of body weight (Figure S12). Overall, selectively inhibiting calpain 2 with CNa **29** is a potent strategy for TNBC therapy (Figure S13).

## 3 Discussion

Calpain 2, a calcium‐dependent cysteine protease, exerts a wide‐ranging impact on tumorigenesis, cancer progression, tumor metastasis, and chemoresistance through the regulation of substrate protein activities and degradation [40]. In humans, there are currently 15 isoforms of calpain [41, 42], with calpain 1 and 2 being ubiquitously expressed. Several studies focusing on calpain 2 have implicated its expression and activity as crucial factors positively associated with cancer biology across various cancer types. Calpain 2 has been linked to the AKT signaling pathway in conditions such as castration‐resistant prostate cancer [43], renal cell carcinoma [16], and non‐small cell lung cancer [44, 45]. It has also been found to regulate the β‐catenin signaling pathway in hepatocellular carcinoma (HCC) [46] and pancreatic cancer [47]. Moreover, several factors have been identified as regulators of calpain 2 activity and expression in cancers. Studies have shown that LIPH [48], talin‐1 [49], and circRILPL1 [50] contribute to the elevation of calpain 2 expression, while miR‐124 [51] inhibits it. Notably, PTP1B, activated by calpain 2‐mediated cleavage, dephosphorylates SRC at Y530, enhancing the proteolytic activity of calpain 2 by phosphorylating its Y625 in HCC [46]. These previous studies highlighting the upregulation of calpain 2 in cancers underscore its potential as a promising therapeutic target in cancer treatment. However, despite the involvement of calpain 2 in cancer biology, most studies failed to address specificity against different calpain isoforms or to provide insight into strategies for regulating isoform‐specific activity.

In the context of TNBC, a previous study reported on the levels of calpain system proteins, notably demonstrating a significant correlation of prognosis with calpain 2 expression rather than calpain 1 or calpastatin expression. Calpastatin is an endogenous pan‐inhibitor of calpains [18]. In our study, we intended to address the underlying mechanism of the correlation between calpain 2 and the clinical outcome of TNBC patients. The isoform‐specific involvement of calpain 2 in clinical outcomes (Figures 1A–C and S1) and mesenchymal properties in TNBC (Figures 1D–F and S2) was elucidated through diverse database analyses, and *CAPN1* or *CAPN2* modulated TNBC cells (Figures 2, 3, 4). Microarray data of TNBC cell lines showed that basal B type cells, characterized by higher levels of mesenchymal markers, tend to express a higher level of *CAPN2* and a lower level of *CAPN1* compared to basal A type cells (Figure 1D,E). The developed *CAPN2* knockdown cells exhibited distinct phenotypical changes compared to *CAPN1* knockdown cells. Experimental results evaluating migration and invasion capacity, as well as cellular proliferation, suggested distinct roles of the two major isoforms of calpain (Figures 2 and S4). Moreover, in GSEA analysis, *CAPN2* expression showed a significant correlation with the EMT hallmark gene set, whereas *CAPN1* expression showed a negative correlation with the same gene set (Figures 3A,B, and S14).

EMT, a pivotal factor governing the metastatic phenotype, is activated by oncogenes, transcription factors, cytokines, and stressors such as hypoxia [52]. Hypoxia, characterized by low oxygen concentrations, is a prevalent condition in most solid tumors [53]. This condition arises from insufficient oxygen supply, which fails to meet the demands of rapidly growing cancer tissue and often does not reach the inner tumor regions [53]. The induction of EMT by hypoxia is proposed due to the interconnection between the signaling pathways. Among hypoxia‐related signaling, HIF1α has been reported to closely associate with EMT pathways by regulating various EMT transcription factors, such as Twist, Snail, Slug, SIP1, and ZEB1, through direct and indirect mechanisms [53]. In this study, we confirmed that calpain 2 regulates the nuclear localization and activity of HIF1α, which dictates *TWIST1* transcription, controlling the EMT process (Figures 3, 4, and S5–S8). For assessment of the tumor microenvironment, HIF1α levels were also evaluated in tumor spheroids (Figure S8).

Calpain‐mediated filamin A cleavage has been reported to control HIF1α activity in several cancer cell lines, including melanoma, osteosarcoma, and cervical cancer cells. [29]. Calcium has also been implicated in regulating filamin A cleavage, which is associated with migration in prostate cancer cells [54]. Blocking calpain‐induced cleavage of filamin A has been shown to decrease proliferation, migration, and colony formation in several types of cell lines, such as human melanoma, human prostate cancer, mouse fibrosarcoma, and mouse pancreatic cells [55]. Previous reports have demonstrated calpain involvement in HIF1α activation using the calpain inhibitor calpeptin, which inhibits both calpain 1 and 2 [29, 55]. However, previous studies did not determine the isoform‐specific involvement of calpain in filamin A cleavage and related metastatic molecular alterations. Furthermore, hypoxia‐induced HIF1α activation promotes calpain 2, leading to the enzymatic cleavage of talin‐1 and β1 integrin, which facilitates amoeboid migration of breast and head and neck cancer cells [56]. As such, hypoxia and calpain are intricately related to each other in various types of cancer biology. Building upon these findings, our study demonstrates that calpain 2 can modulate the activity of HIF1α in an isoform‐specific manner and may serve as a prognostic factor in TNBC. Calpain 2, rather than calpain 1, regulates the EMT process and metastatic phenotype through calpain 2‐specific filamin A cleavage, followed by HIF1α nuclear translocation and transcriptional activation (Figure 4C,E). Considering the elevation in intracellular calcium levels under hypoxic conditions and in the inner region of tumor spheroids (Figure S9) [57, 58], hypoxia can activate both calpain 1 and 2, the major isoforms of calpain. However, according to our results, specificity in filamin A cleavage determines the involvement in the HIF1α‐induced EMT process. This suggests that calpain 2‐specific inhibition can effectively control metastasis induced by hypoxic conditions.

The close correlation between calpain 2 expression and clinical outcomes was observed exclusively in the TNBC subtype (Figures 1A and S1A). This TNBC‐specific correlation appears to be linked to the relative level of filamin A. While HIF1α‐regulating factors are diverse, filamin A level is higher in TNBC compared to other subtypes of breast cancer, and filamin A cleavage is a critical factor in HIF1α activity in TNBC (Figure S15).

Calpain inhibitors have been studied and developed for decades. However, there is not a clinically available drug. The primary challenges in calpain inhibitor development include low selectivity against other cysteine proteases or calpain isoforms and structural instability due to peptidomimetic structures or aldehyde functional groups, resulting in susceptibility of the inhibitors to metabolism in physiological conditions [40, 59]. To overcome these hurdles, we employed a non‐peptidomimetic and isoform‐selective calpain inhibitor, CNa **29**, to explore the clinical implications of pharmacological calpain 2 inhibition. Treatment with CNa **29** significantly impeded filamin A cleavage, the EMT process, and *TWIST1* expression and decreased metastatic incidence (Figures 6 and S14).

Doxorubicin is one of the most frequently used drugs in cancer therapy for various types of cancer [60]. In the context of TNBC, doxorubicin is employed as first‐line therapy, especially in certain types of TNBC lacking newly identified therapeutic targets such as PD‐L1 and BRCA mutation [61]. However, despite its widespread use, long exposure to doxorubicin may lead to acquired drug resistance [62]. Emerging evidence suggests that cancer stemness and EMT are involved in doxorubicin resistance [63, 64, 65]. An increase in cancer cell stemness and mesenchymal properties contributes to recurrence, distant metastasis, and drug resistance. Calpain 2 inhibition effectively reversed EMT and attenuated stem cell‐like properties (Figures 3 and S10). In addition, CNa **29** showed a synergistic enhancement of anti‐cancer activity when combined with doxorubicin (Figure S12). Moreover, a previous study of doxorubicin resistance exhibited calpain 2 involvement in the mechanism of resistance development in an isoform‐specific manner [66]. Collectively, pharmacological calpain 2‐selective inhibition using CNa **29** may improve the effectiveness of therapeutic modalities of doxorubicin and overcome the development of drug resistance in TNBC.

Recent research on TNBC has been actively focusing on drug resistance and metastasis [11, 67, 68], as these two factors are crucial determinants of clinical outcomes. In this study, we explored the role of calpain 2 in TNBC drug resistance and metastasis and the underlying mechanisms. Calpain 2 plays a role in EMT‐mediated metastasis through the regulation of HIF1α, which may serve as a regulatory mechanism for metastasis to various organs, not just the lung. Given that TNBC metastasis is influenced by distinct regulatory mechanisms depending on the target organ, further studies are needed to examine metastasis to different organs, such as the liver, or brain. In addition, as our findings are specific to TNBC models, further research is required to evaluate the efficacy of calpain 2 inhibition as a therapeutic strategy in other cancer types to assess its broader applicability.

Our study elucidated an unrecognized isoform‐specific role of calpain 2 in TNBC. We discovered that calpain 2‐specific cleavage of filamin A enhances the nuclear localization of HIF1α, inducing the EMT through *TWIST1* expression. This heightened EMT process amplifies proliferation, migration, and invasion capacity, as well as metastasis in an in vivo metastatic model. Moreover, the non‐peptidomimetic calpain 2‐selective inhibitor, CNa **29**, demonstrated a significant attenuation of cancer metastasis, accompanied by downregulation in proliferation, migration, and the EMT (Figure S14). Given that previously discovered calpain inhibitors have limitations due to their peptidomimetic structure and lack of isoform selectivity, the therapeutic potential of CNa **29** in TNBC is significant. We propose that therapeutic intervention of calpain 2 isoform‐selective inhibition represents a promising strategy for preventing metastasis and treating this challenging type of cancer, TNBC.

## 4 Materials and Methods

### 4.1 Bioinformatic Analysis

For survival analysis, the Kaplan–Meier plotter (http://kmplot.com/breast/) was used [69]. *CAPN2* or *CAPN1* gene (probe set 208683_at or 200752_s_at) was analyzed, and results were displayed as a Kaplan–Meier survival plot. *CAPN2* gene expression was analyzed using univariate Cox proportional analysis and correlation with mesenchymal genes using b‐GenExMiner [70, 71] and TIMER [72]. Gene expression assessment in cancer cell lines was conducted using Cancer Cell Line Encyclopedia (CCLE) microarray data (GSE36133) [73]. GSEA was investigated using GSE103091, a dataset of gene expression in TNBC patients [74, 75].

### 4.2 Cell Culture

TNBC cell lines (MDA‐MB‐231, MDA‐MB‐436, and Hs578T) were cultured at 37°C in a humidified atmosphere with 5% CO_2_. MDA‐MB‐231 cells were cultured in RPMI (Welgene, Korea) containing 10% fetal bovine serum (FBS, Hyclone, USA), while MDA‐MB‐436 and Hs578T cells were cultured in DMEM (Welgene, Korea) containing 10% FBS. Cell lines were obtained from the Korean Cell Line Bank. The MDA‐MB‐231‐luc cell line was used in in vivo metastasis studies. Luciferase activity was tested before cell injection into the tail vein of in vivo models.

### 4.3 Quantitative Real‐Time Polymerase Chain Reaction (qRT‐PCR)

Total RNA was extracted using TRIzol reagent (Invitrogen, USA). Reverse transcription of mRNAs was performed using the PrimeScript RT Reagent Kit (Takara, USA). qRT‐PCR was performed using the SensiFAST SYBR No‐ROX Kit (Bioline, Korea). GAPDH and U6 were used as internal controls, and the relative mRNA expression was detected using the CFX96 RT‐PCR detection system (Bio‐Rad, Korea) at the EWHA drug development research core center and the 2^−ΔΔCT^ method. Primer sequences used in this study are listed in Table S1.

### 4.4 Western Blot

Proteins were extracted using RIPA buffer and measured using a Pierce BCA Protein Assay Kit (Thermo Fisher Scientific, USA). Proteins were isolated using 10%–15% SDS‐PAGE and subsequently transferred to PVDF membranes (PALL Corp., USA). Membranes were blocked with 5% skimmed milk or BSA and incubated overnight with primary antibodies. The membranes were then washed and incubated with HRP‐labeled secondary antibodies for 60 min at room temperature. The immune response bands were visualized using enhanced chemiluminescence (Amersham ECL Prime, GE Healthcare, USA; ChemiDoc MP, Bio‐RAD, USA). All antibody information is shown in Table S2.

### 4.5 Immunofluorescence

Cells were seeded in eight‐well chamber slides (SPL, Korea). After reaching 80% confluency, cells were washed with PBS, fixed with paraformaldehyde, and blocked with blocking solution containing 5% Blocking One‐P (Nacalai Tesque, Japan) and 0.1% Triton‐X in PBS. Primary antibodies were applied overnight at 4°C, followed by secondary antibodies for 1 h at room temperature. DAPI was used for nuclei staining. The cells were then washed three times, treated with mounting solution (Dako, Agilent Pathology Solutions, USA), and covered with a cover glass. Images were obtained using an apotome laser‐scanning microscope at the EWHA drug development research core center and were analyzed with Zen Pro software.

### 4.6 Chromatin Immunoprecipitation (ChIP) Assay

Cells were seeded in a 150 mm cell culture plate (Nunc, USA) and cultured until reaching approximately 80% confluency. To assess HIF1α binding to the HRE of the *TWIST1* promoter, cells were cultured in serum‐free medium for 21 h, followed by an additional incubation for 3 h in serum‐free medium containing 50 µM CoCl_2_ prior to the ChIP assay. The ChIP assay was conducted using the Pierce Agarose ChIP Kit (Thermo Fisher Scientific, USA) according to the manufacturer's instructions. The HIF1α‐bound HRE of *TWIST* was evaluated using qRT‐PCR.

### 4.7 In Vivo Metastasis Study

Five‐week‐old female BALB/c nude mice were procured from Orient Bio (Korea). Animal handling was conducted in accordance with ethical guidelines approved by the Animal Experiment Ethics Committee of Ewha Womans University, adhering to relevant regulations (IACUC20‐032, IACUC23‐066). Throughout the study, mice had unrestricted access to food and water and were maintained under constant temperature and humidity with a 12 h light/dark cycle.

MDA‐MB‐231‐luc cells (2 × 10^5^) were injected into the tail vein of the mice. Metastatic incidence and body weight were evaluated every 2–3 days. Metastatic assessment was conducted using the in vivo imaging system (IVIS) Lumina Series III (Perkin Elmer, USA) at the EWHA drug development research core center. Metastasis was measured after luciferin injection. The intensity of luminescence was quantified in the same area of the region of interest (ROI) with 60 s exposure.

To evaluate anti‐metastatic activity, CNa **29** was administered intraperitoneally at doses of 15 or 30 mg/kg daily, starting the day after cell injection. In the experiment assessing metastatic inhibition by CNa **29**, the body weight of each mouse was measured daily.

### 4.8 Statistical Analysis

Statistical analysis was performed using GraphPad Prism 10.0.0 software (GraphPad Software, USA). Data are presented as mean ± standard error of the mean (SEM). The two‐tailed Student's *t*‐test was used for comparisons between two groups, and one‐way or two‐way ANOVA followed by a multiple comparison test. A *p* value less than 0.05 (*p* < 0.05) was considered statistically significant.

## Author Contributions

Y. K. conceptualized, data curated, supervised the study, revised and validated the manuscript. K‐H. J. conducted the experiments, analyzed the data, data curated, and wrote the draft. S. P., E. S. P., J‐A. K., Y. L., and S‐Y. H. conducted the experiments. Y. N. supervised the study, conducted the experiments, and reviewed the manuscript. All authors read and approved the final manuscript.

## Ethics Statement

Animal handling was conducted in accordance with ethical guidelines approved by the Animal Experiment Ethics Committee of Ewha Womans University, adhering to relevant regulations (IACUC20‐032, IACUC23‐066).

## Conflicts of Interest

The authors declare no conflicts of interest.

## Supporting information



Supporting Information

## Data Availability

The data that support the findings of this study are available from the corresponding author upon reasonable request.
