# Supplementary material for: Calpain 2 Isoform‐Specific Cleavage of Filamin A Enhances HIF1α Nuclear Translocation, Promoting Metastasis in Triple‐Negative Breast Cancer
Source: MedComm (2020). 2025 Mar 27;6(4):e70147. doi: 10.1002/mco2.70147 (PMC11949505; doi:10.1002/mco2.70147)
Supplement: Supplementary file 1 — Supporting Information [file MCO2-6-e70147-s001.docx]

***Supplementary Materials***

**Calpain** **2 Isoform-Specific Cleavage of Filamin A** **Enhances HIF1α Nuclear Translocation, Promoting** **Metastasis in Triple Negative Breast Cancer**

Kyung-Hwa Jeon,^1^ Seojung Park,^1^ Eun Seon Pak,^1^ Jeong-Ahn Kim,^1^ Yi Liu,^1^ Soo-Yeon Hwang,^1^ Younghwa Na^2^ and Youngjoo Kwon^1,*^

*^1^College of Pharmacy, Graduate School of Pharmaceutical Sciences, Ewha Womans University, Seoul, 120-750, Republic of Korea*

*^2^College of Pharmacy, CHA University, 120 Haeryong-ro, Pochon-shi, Gyeongghi-do, 11160, Republic of Korea.*

**Materials and Methods**

**IHC, TMA slide**

The expression levels of Calpain 2 proteins in TMA slide were evaluated using IHC. Paraffin-embedded TMA block was sliced and provided from manufacturer (BR1509, TissueArray.Com, MD, USA). The tissue slices were dried in a dry oven at 70’C? for 2 hr, dewaxed with xylene, and rehydrated with graded ethanol. The slides were then placed in 10 mM citrate buffer (pH6.0) and heated in a microwave oven at high temperature for 5 min. This antigen retrieval process was repeated for 2 to 3 cycles. Endogenous peroxidase was blocked with hydrogen peroxide, and cells ere incubated with 5% BSA for 1 hr to reduce nonspecific staining. Subsequently, the slices were incubated overnight at 4’C with primary antibodies against calpain 2. Immunostaining was performed with a secondary antibody labeled with horseradish peroxidase at 37’c for 1 hr. After applying DAB chromogen, staining was carried out with hematoxylin for 3 min, followed by a 30second blue counterstain. Images were captured using histological microscope, and the percentage of positive area was calculated using Image J software.

**Cell culture**

TNBC cell lines (MDA-MB-231, MDA-MB-436, and Hs578T) were cultured at 37°C in a humidified atmosphere with 5% CO2. MDA-MB-231 cells were cultured in RPMI (Welgene, Korea) containing 10% fetal bovine serum (FBS, Hyclone, USA), while MDA-MB-436 and Hs578T cells were cultured in DMEM (Welgene, Korea) containing 10% FBS. Cell lines were obtained from Korean Cell Line Bank. The MDA-MB-231-luc cell line was used in in vivo metastasis studies. Luciferase activity was tested before cell injection into the tail vein of *in vivo* models.

**WST assay**

Cells were seeded at 10^4^ cells/wll in a 96-well cell culture plate. Cells were incubated for the indicated duration at 37’C in a 5% CO2 incubator. Viability of the cells were evaluated by measuring the absorbance at 450 nm after applying 5 μL of EZ-cytoX to each well. For the measurement, ELISA Microplate Reader (VersaMax, Molecular Devices) was utilized.

**Live cell counting**

Cells were seeded at 105 cells/wll in a 6-well cell culture plate. Cells were incubated for the indicated duration at 37℃ in a 5% CO_2_ incubator. Viable cells were stained with trypan blue diluting at 1:1 ratio and counted using Cell counter.

**Cellular circularity calculation**

Cellular circularity was calculated using ImageJ software (National Institute of Health, USA). Images used in circularity calculation was taken by an apotome laser-scanning microscope (Carl Zeiss, Germany). The cellular morphology was analyzed based on the area and perimeter of detected particles. The circularity was calculated using the equation, circularity = 4 × π (area/perimeter^2^). The 50 particles of each types of cells were analyzed.

**Immunofluorescence**

Cells were seeded in eight-well chamber slides (SPL, Korea). After reaching 80% confluency, cells were washed with PBS, fixed with paraformaldehyde, and blocked with blocking solution containing 5% blocking one-P (Nacalai Tesque, Japan) and 0.1% triton-X in PBS. Primary antibodies were applied overnight at 4 ℃, followed by secondary antibodies for 1 h at room temperature. DAPI was used for nuclei staining. The cells were then washed three times, treated with mounting solution (Dako, Agilent Pathology Solutions, USA), and covered with a cover glass. Images were obtained using an apotome laser-scanning microscope at EWHA drug development research core center and were analyzed with Zen pro software.

**Chromatin immunoprecipitation (ChIP) assay**

Cells were seeded in a 150 mm cell culture plate (Nunc, USA) and cultured until reaching approximately 80% confluency. To assess HIF1α binding to the hypoxia response element (HRE) of TWIST1 promoter, cells were cultured in serum-free medium for 21 h, followed by an additional incubation for 3 h in serum-free medium containing 50 μM CoCl2 prior to the ChIP assay. The ChIP assay was conducted using the Pierce™ Agarose ChIP kit (Thermo Fisher Scientific, USA) according to the manufacturer’s instructions. The HIF1α-bound HRE of TWIST was evaluated using qRT-PCR.

**Supplementary Table S1. The nucleotide sequences of the primers used for qPCR**

| Target | Forward | Reverse |
| --- | --- | --- |
| CDH1 | CGACCCAACCCAAGAATCTA | AGGCTGTGCCTTCCTACAGA |
| TJP1 | GAACGAGGCATCAATCCCTAA | CCAGCTTCTCGAAGAACCAC |
| VIM | GAGAACTTTGCCGTTGAAGC | TCCAGCAGCTTCCTGTAGGT |
| FN1 | CAGTGGGAGACCTCGAGAAG | GTCCCTCGGAACATCAGAAA |
| TWIST1 | GTCCGCAGTCTTACGAGGAG | TGGAGGACCTGGTAGAGGAA |
| TWIST1-HRE | CGGGGGAGGGGGACTGGAAAGC | AGGCCTCCTGGAAACGGTGCCG |
| SNAI1 | TAGGGGTGCTGGAAGGTAAA | GGTTCTTCTGCGCTACTGCT |
| SNAI2 | GCATTTCTTCACTCCGAAGC | TGAATTCCATGCTCTTGCAG |
| SMAD4 | TTGCTTCCACTTGAATGCTG | CTTCAAAGGGGACACCAAAA |

**Supplementary Table S2. The sources of antibodies used in this study**

| Target | Company | Catalog No. | Application |
| --- | --- | --- | --- |
| Calpain 2 | Cell Signaling Technology | 2539S | IHC, WB |
| Calpain 1 | Cell Signaling Technology | 2556S | WB |
| α-Tubulin | MBL Life science | M175-3 | WB |
| GAPDH | Santa Cruz Biotechnology  MBL Life science | SC32233  MBL171-3 | WB |
| β-Actin | Cell Signaling Technology | 4967S | WB |
| E-cadherin | BD | 610182 | WB |
| Vimentin | Santa Cruz Biotechnology | SC-6260 | WB, IF |
| HIF1α | Cell Signaling Technology | 14179S | IF, ChIP |
| Filamin A | EMD Millipore Corp | MAB1680 | WB |
| Smad4 | Thermo scientific | MAS-15682 | WB |
| Slug | Santa Cruz Biotechnology | SC-166476 | WB |
| Snail | Santa Cruz Biotechnology | SC-393172 | WB |
| phospho-MAPK | Cell Signaling Technology | 9101S | WB |
| phospho-AKT | Santa Cruz Biotechnology | SC-7985 | WB |
| Lamin A/C | Cell Signaling Technology | 2032S | WB |
| Flag | MBL Life science | M185-3L | WB |
| Nanog | Cell Signaling Technology | 4903S | WB |
| Oct4 | Cell Signaling Technology | 2750S | WB |
| Sox2 | Cell Signaling Technology | 3579S | WB |

**Supplementary Table S3**


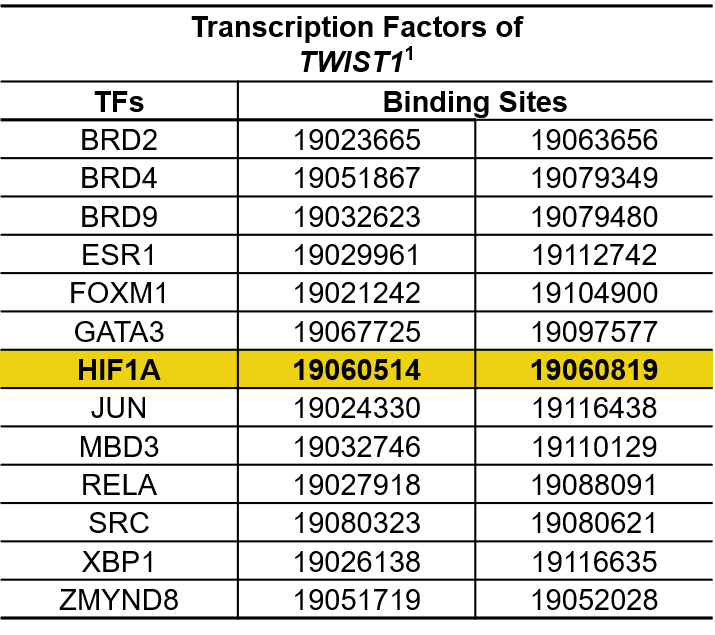


^1Transcription factors binds near^ *^TWIST1^* ^gene (max gene distance, 100) were predicted using GTRD database.^


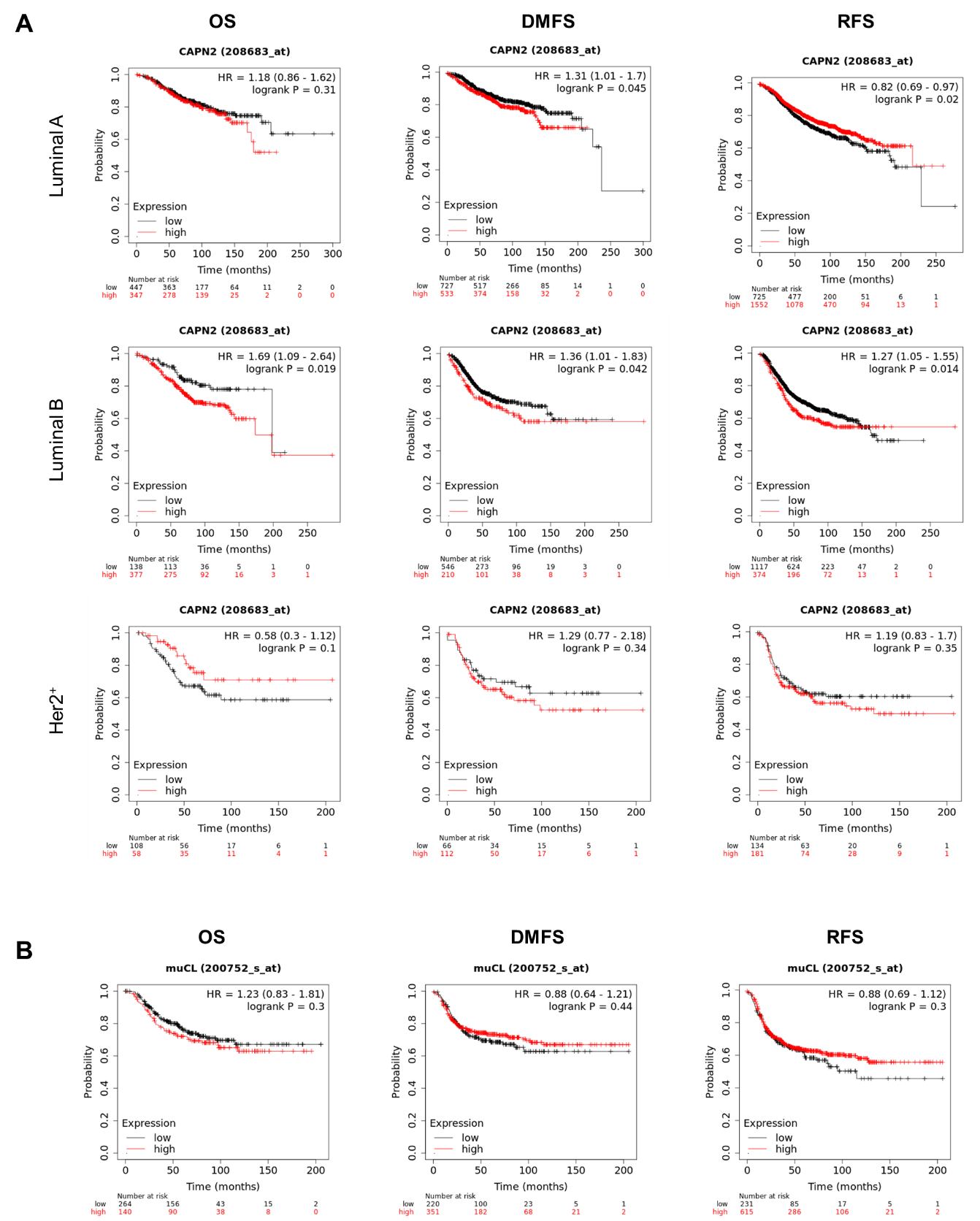


**Figure S1. The correlations between clinical outcomes and CAPN expressions. (A)** The correlations between clinical outcomes and CAPN2 expression in each subtype of breast cancer. **(B)** The correlation between clinical outcomes and CAPN1 expressions in Triple negative breast cancer.


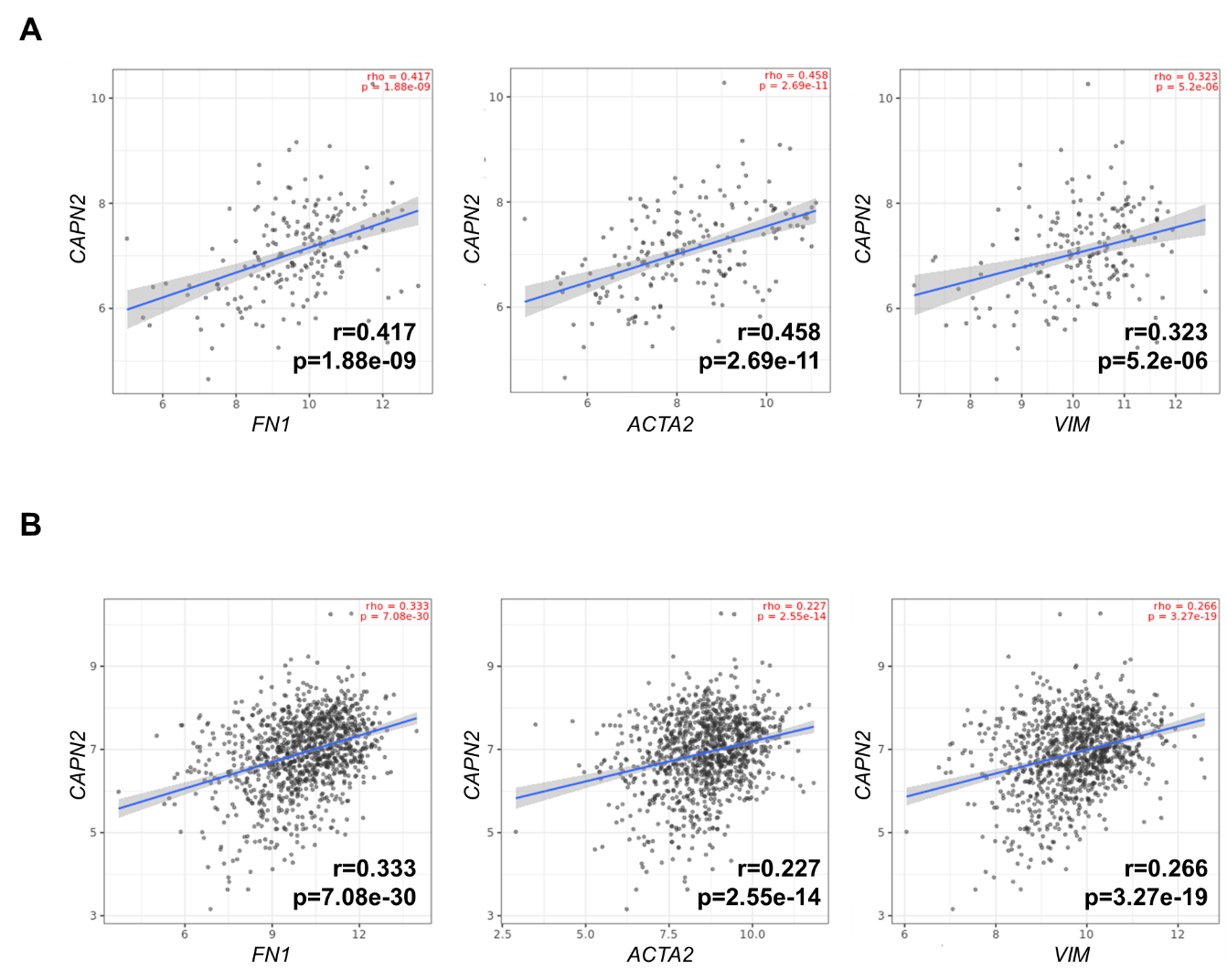


**Figure S2 Correlation plots of *CAPN2* and mesenchymal genes, such as *FN1*, *ACTA2*, or *VIM* in TNBC (A) and in other subtypes of breast cancer (B).** Correlation analysis was conducted using web-based application, TIMER (http://cistrome.org/TIMER/).


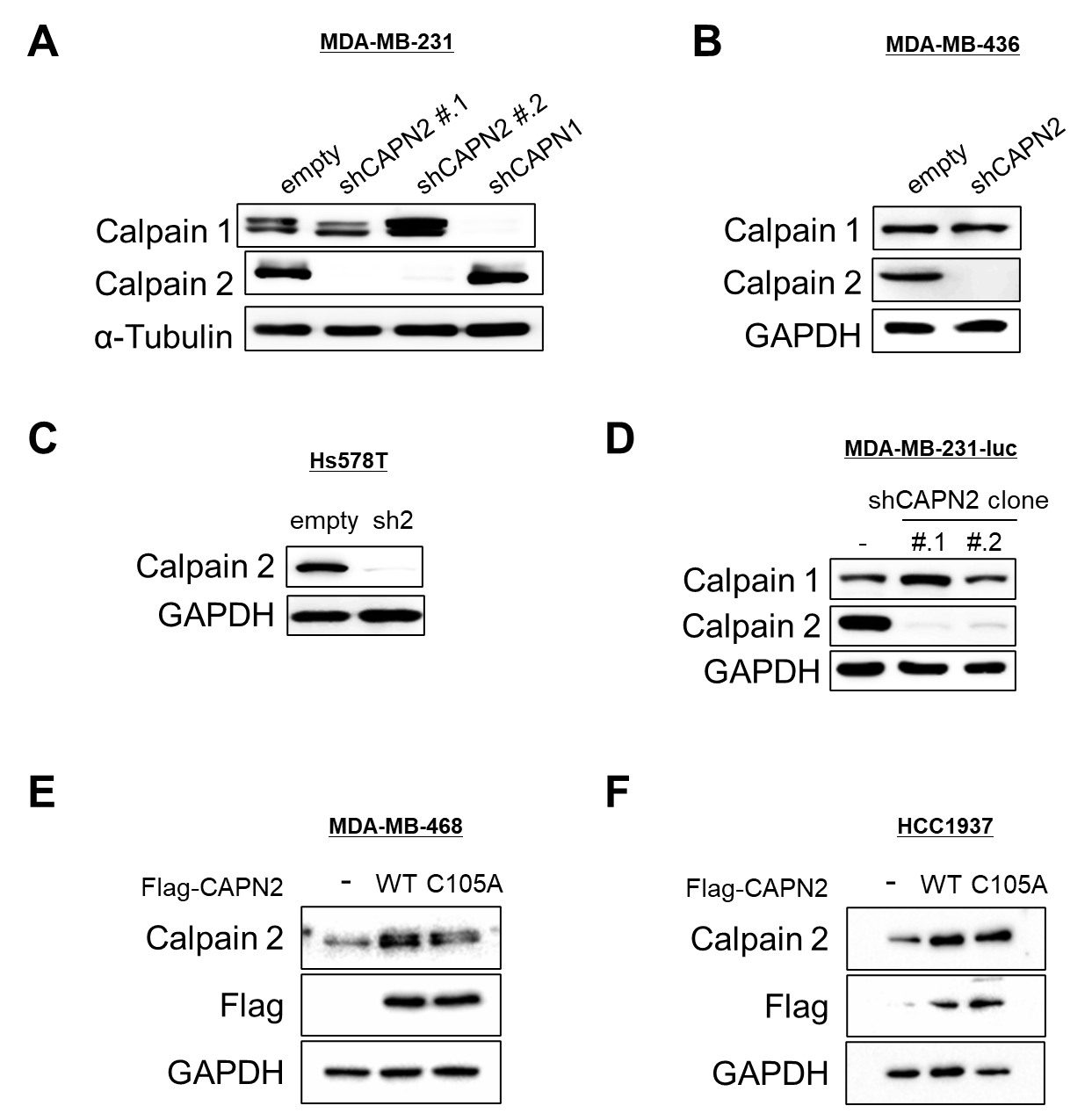


**Figure S3 Development of calpain-regulated TNBC cellular models.** Western blot analysis of control and CAPN1/2 knockdown mesenchymal TNBC cell lines, MDA-MB-231 (A) and MDA-MB-436 (B), Hs578T (C), and MDA-MB-231-luc (D). Overexpression in wild-type or mutated (C105A) CAPN2 in epithelial TNBC cell lines, MDA-MB-468 (E) and HCC1937 (F).


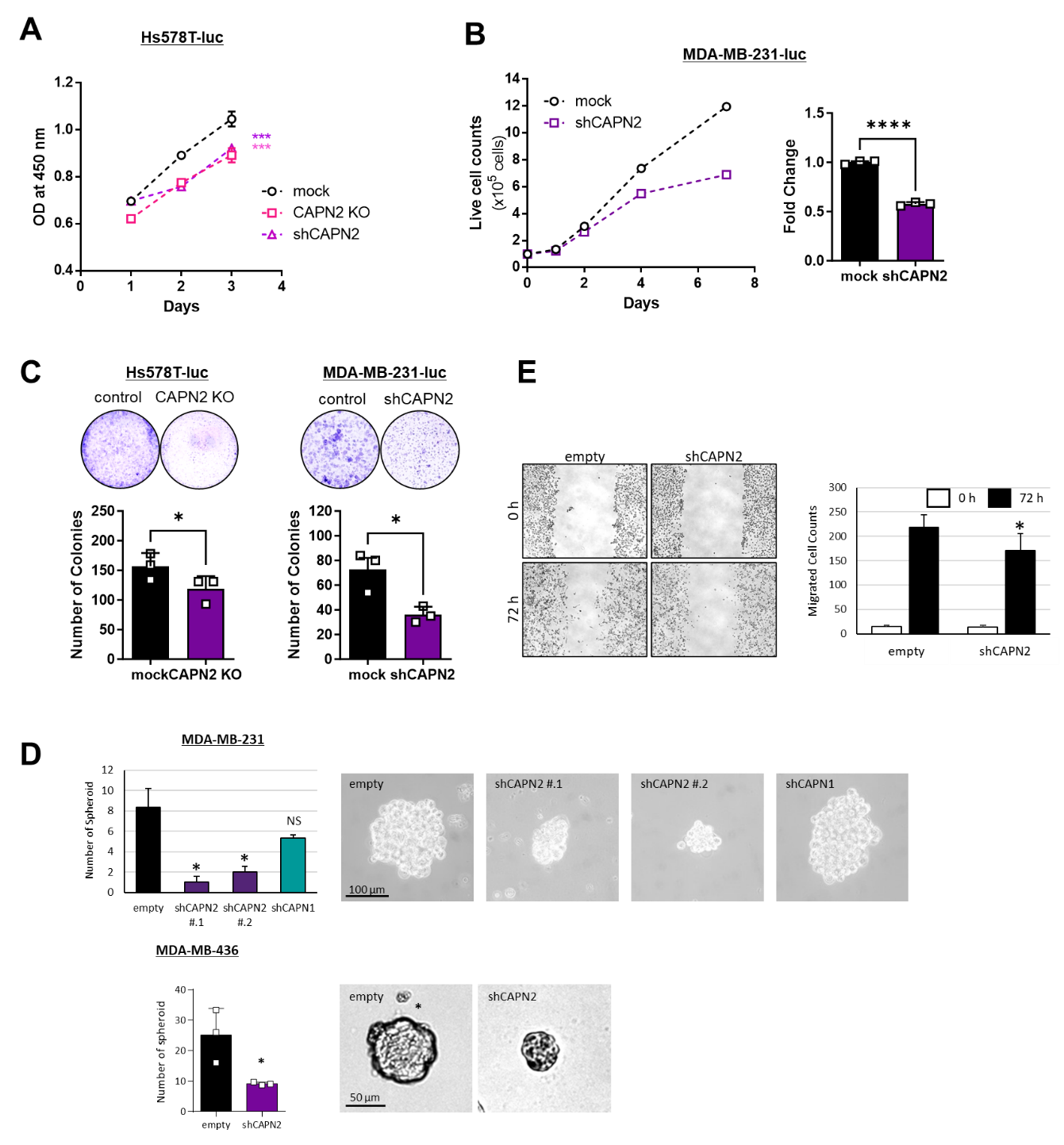


**Figure S4 Phenotypical changes in calpain 2 downregulated cells. (A)** Growth inhibition induced by calpain 2 downregulation was evaluated through WST assay in Hs578T-luc cells. (**B)** Actual live cells were counted after CAPN2 knockdown in MDA-MB-231-luc cells. (**C)** Colony forming ability was assessed in Hs578T-luc and MDA-MB-231-luc cells. (**D)** Growth of colonies under suspension culture system was examined in MDA-MB-231 and 436 cells. (**E)** Cellular migration was evaluated in wound healing assay of MDA-MB-231 cells.


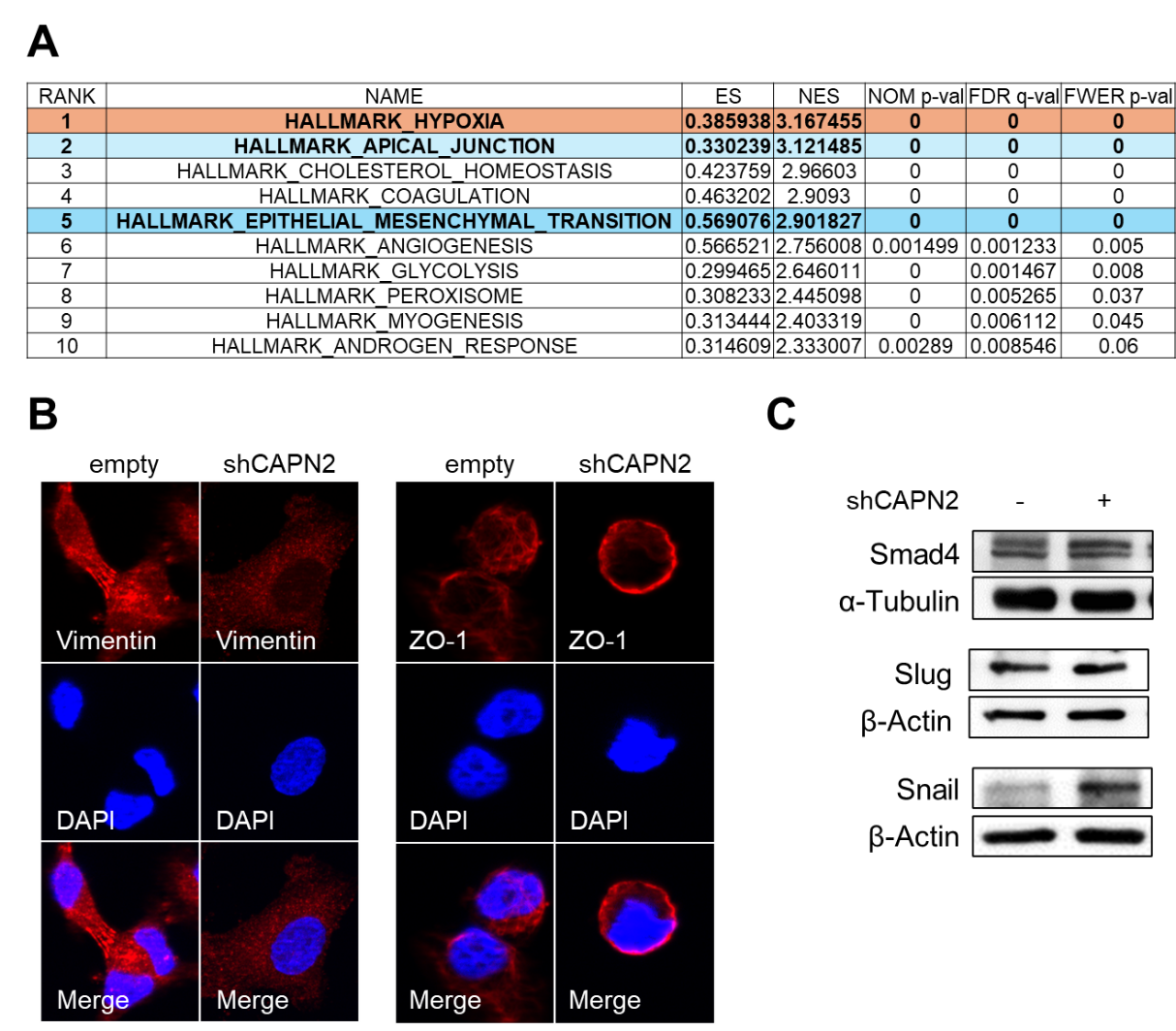


**Figure S5. CAPN2 knockdown affected EMT process. (A)** GSEA results in hallmark examinations. (**B)** Protein levels of vimentin (mesenchymal marker) and ZO-1 (epithelial marker) were analyzed in MDA-MB-231. (**C)** Expression levels of EMT related transcription factors were assessed in MDA-MB-231.


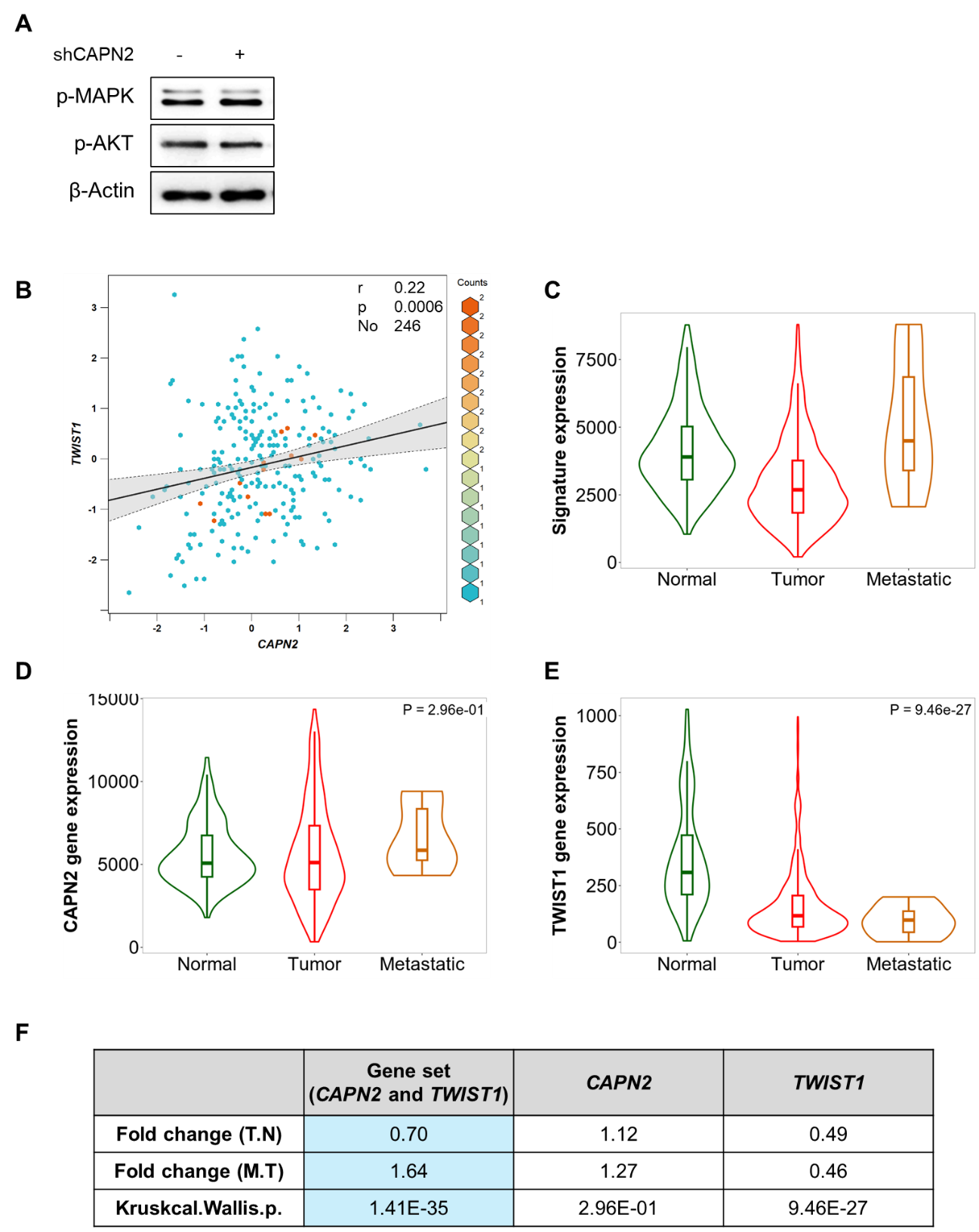


**Figure S7 Regulation of *TWIST1* expression and its correlation with *CAPN2* expression. (A)** Phosphorylated MAPK and AKT levels were assessed in *CAPN2* knockdown MDA-MB-231 cells. (**B)** Scatterplot represents correlation of *CAPN2* and *TWIST1* expressions in basal-like (PAM50) and TNBC (IHC) patients, while the straight line represents linear regression model. Correlation analysis was conducted using web-based application, bc-GenExMiner v.5.0 (<http://bcgenex.ico.unicancer.fr/>). (**B-D)** The mean expression of the gene set (*CAPN2* and *TWIST1*) (B), *CAPN2* (C), or *TWIST1* (D) in adjacent normal breast tissues, breast cancer tissues, and breast cancer metastatic tissues. Data analysis was conducted using web-based database, TNMplot database. **(E)** Fold changes in expression of the gene set, *CAPN2*, and *TWIST1* were analyzed. The gene set of *CAPN2* and *TWIST1* showed the most significant increase in fold change of metastatic to primary tumor.


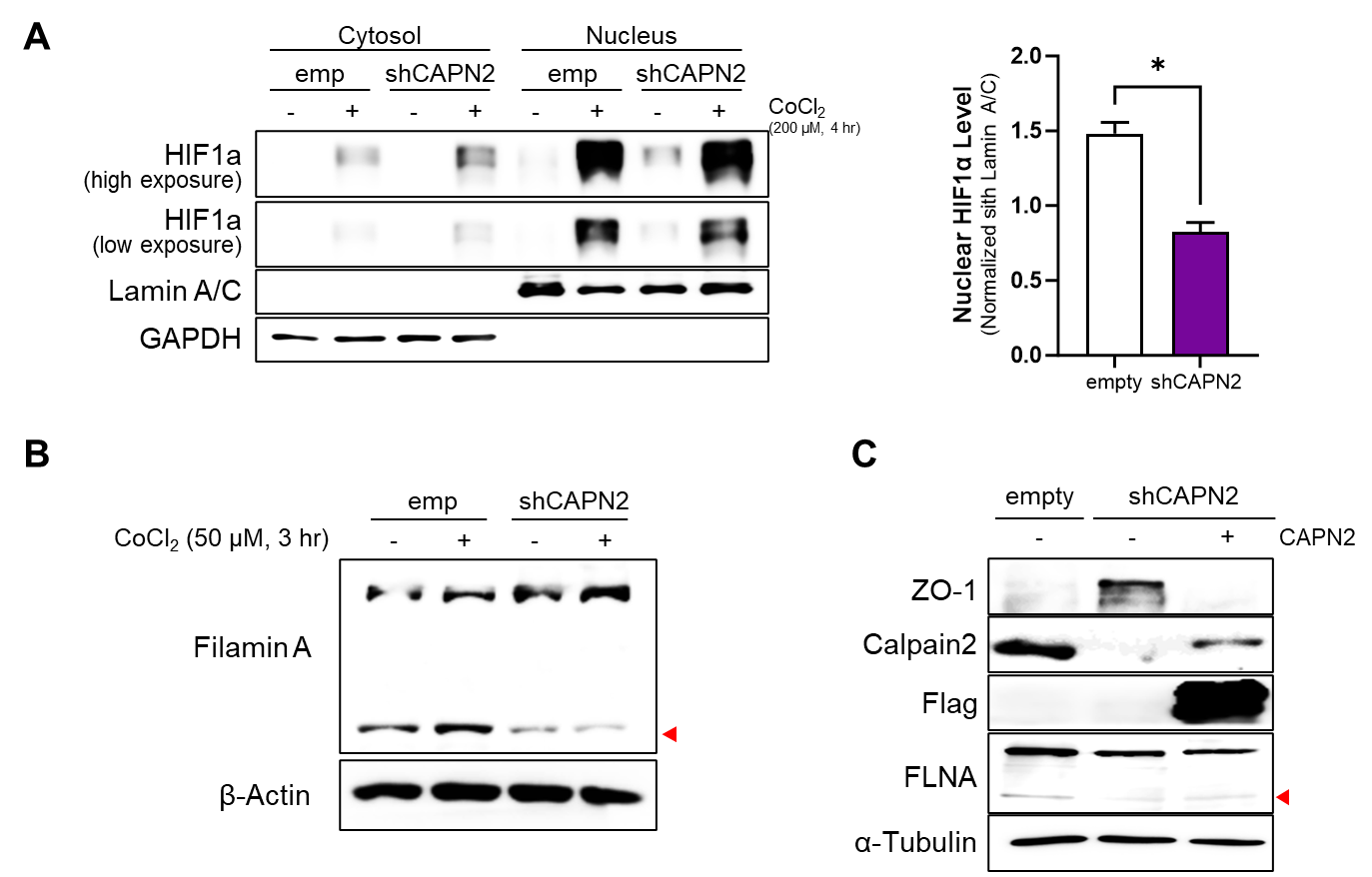


**Figure S7. Calpain 2 downregulation-induced inhibition of HIF1α nuclear localization and filamin A cleavage. (A)** Subcellular localization of HIF1a in CAPN2 knockdown MDA-MB-231 cells. HIF1a translocation to the nucleus was evaluated with or without treatment of CoCl_2_. **(B)** Filamin A cleavage in CAPN2 knockdown MDA-MB-231 cells. Filamin A cleavage was assessed with or without treatment of CoCl_2_. **(C)** ZO-1 expression and filamin A cleavage in CAPN2 knockdown and re-expressed MDA-MB-231 cells.


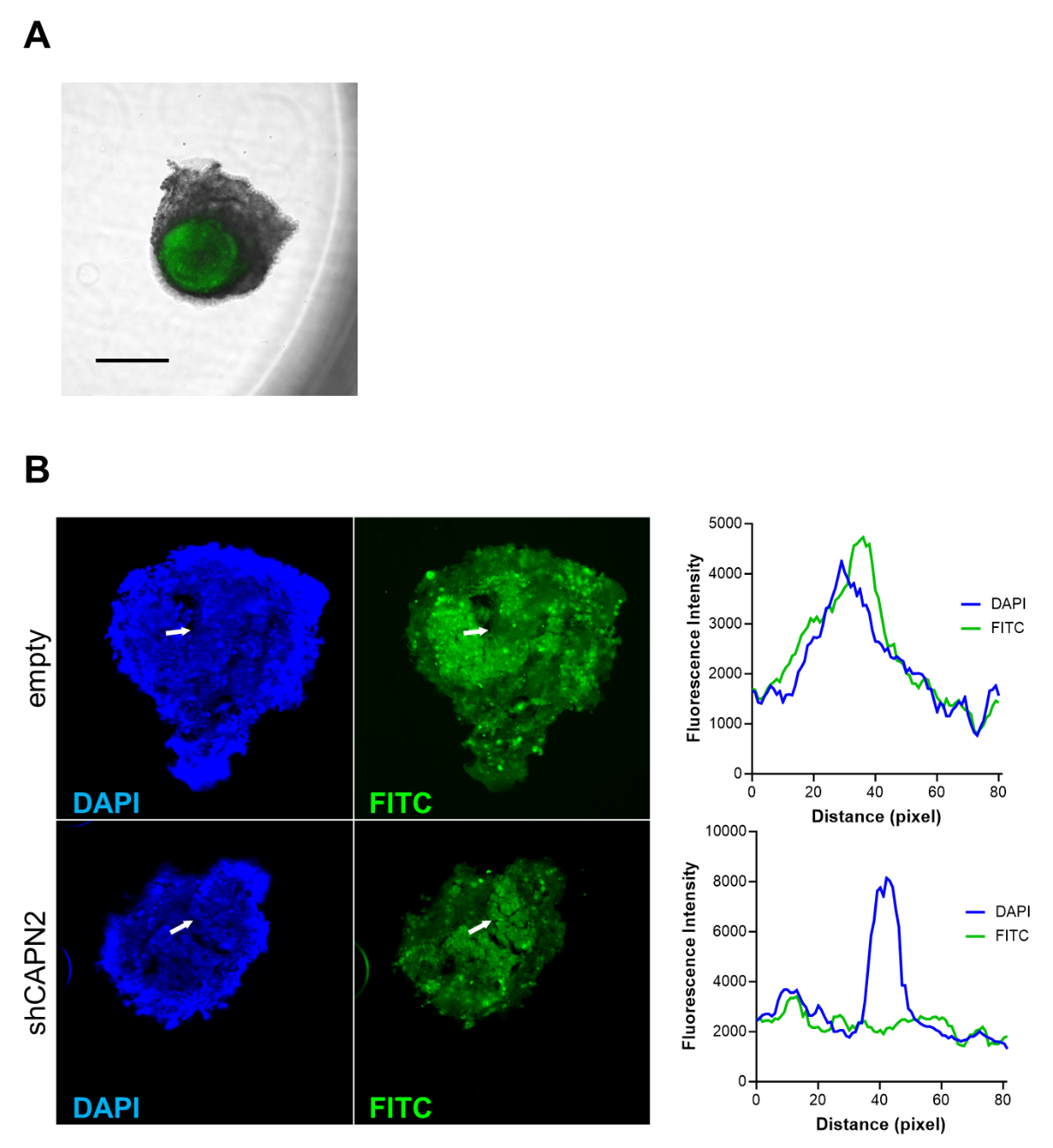


**Figure S8. HIF1α expression and intracellular calcium levels in tumor spheroids of MDA-MB-231 cells. (A)** Intracellular calcium levels detected by Fura-2-AM. **(B)** Comparison of co-localization of HIF1α (FITC) and DAPI signals.


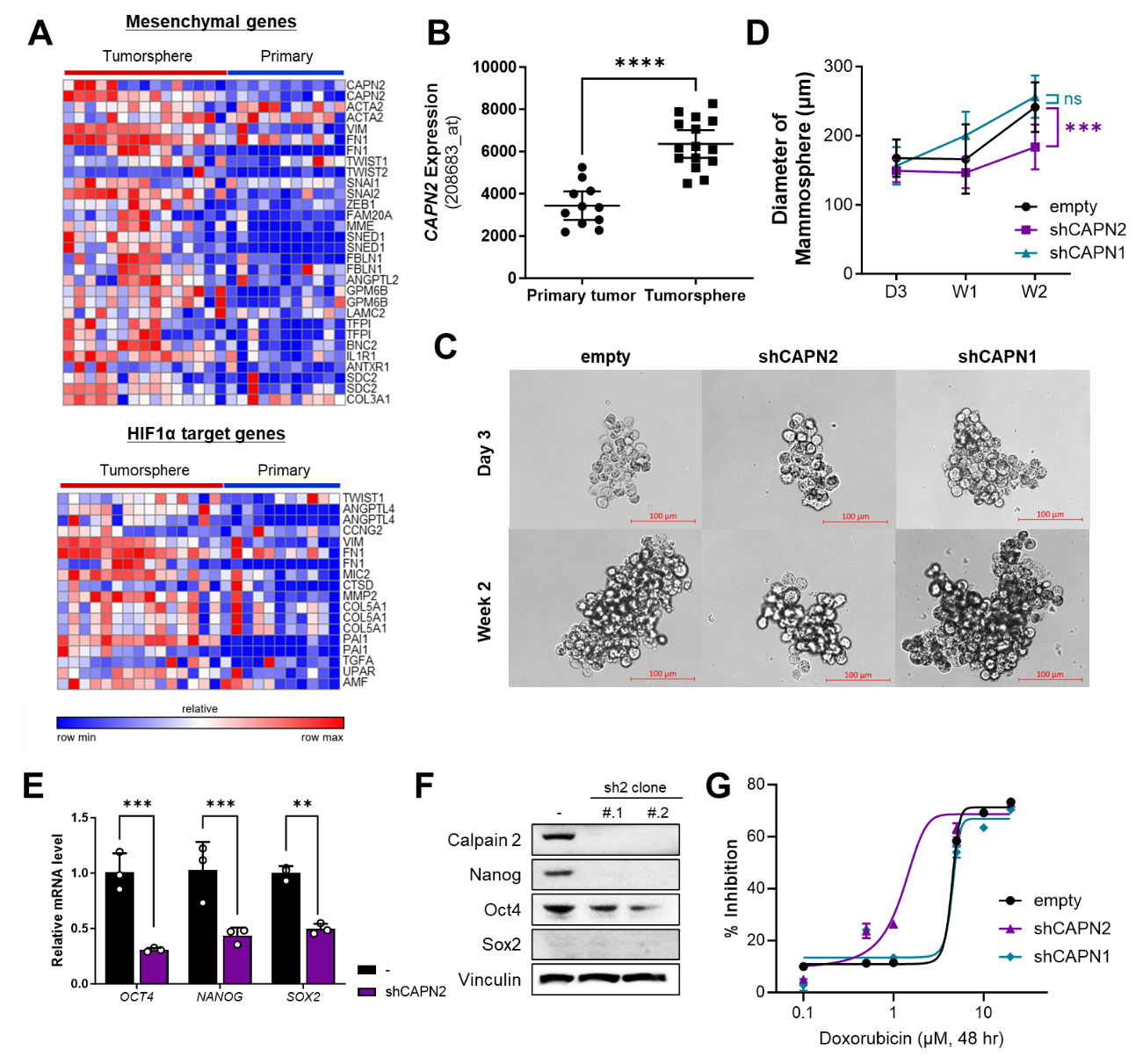


**Figure S9. Calpain 2 expression is required to sustain cancer stemness. (A)** Heatmaps showing mesenchymal and HIF1α target gene expressions in tumorspheres and matched primary tumors (GSE7515). **(B)** *CAPN2* expression of each group. The gene expression values were derived from a published microarray dataset. The data were log2-transformed and mean centered. Primary tumor, n = 11; tumorsphere, n = 15. **(C&D**) Growth of mammosphere of each group of MDA-MB-231. Growth rate of mammosphere was calpain 2 specifically regulated. **(E&F)** Expressions of Nanog, Oct4, and Sox2 in cells stably expressing shCAPN2 evaluated in mRNA (E) and protein levels (F) in MDA-MB-231. **(G)** Drug sensitivity of doxorubicin assessed in MDA-MB-231 cells.


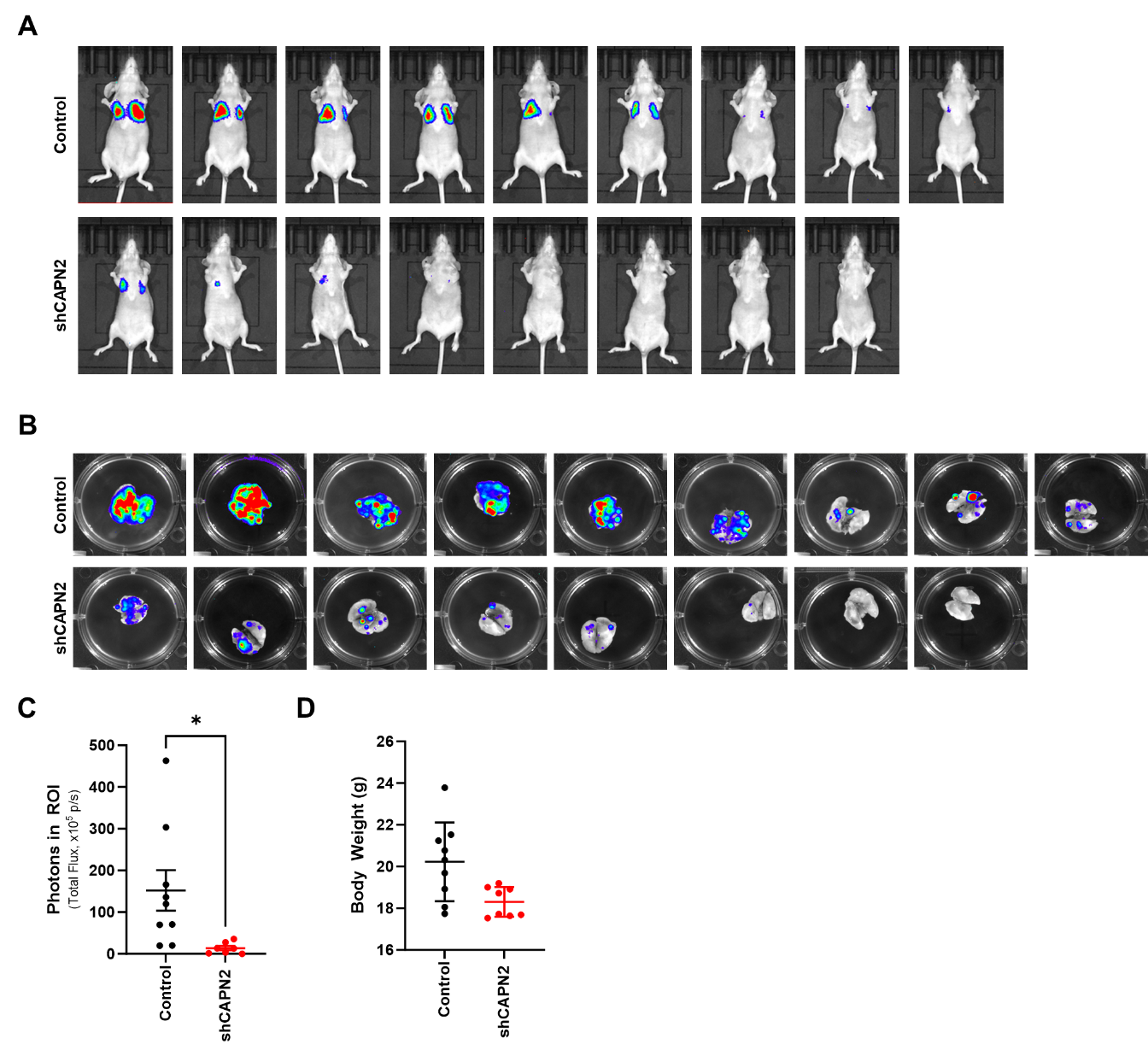


**Figure S10. Attenuation of lung metastasis by calpain 2 inhibition in an in vivo metastasis model. (A)** Bioluminescent images showing metastatic tumors in different groups. **(B)** Bioluminescent ex vivo images showing metastatic tumors in different groups. **(C)** Quantification of bioluminescent ex vivo images. **(D)** Comparison of body weight between groups.


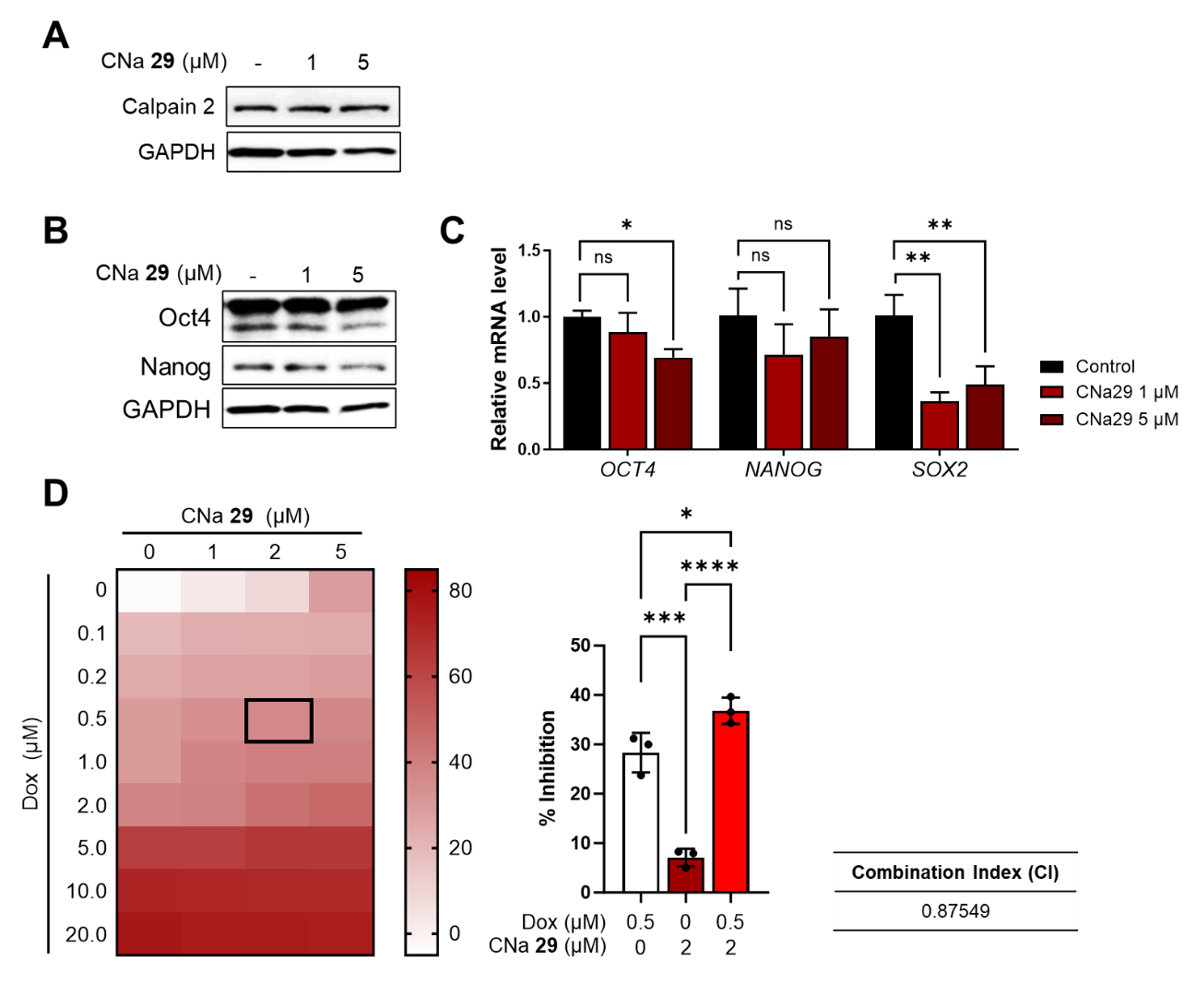


**Figure S11. Inhibitory activity of CNa 29 on cancer stemness in MDA-MB-231 cells. (A)** Constant level of calpain 2 protein under CNa **29** treatment. **(B&C)** Expressions of Nanog, Oct4, and Sox2 in CNa **29** treated cells. **(D)** Drug sensitization and synergistic effect of doxorubicin with CNa **29**.

**Figure S12. Comparison of body weight between groups.**


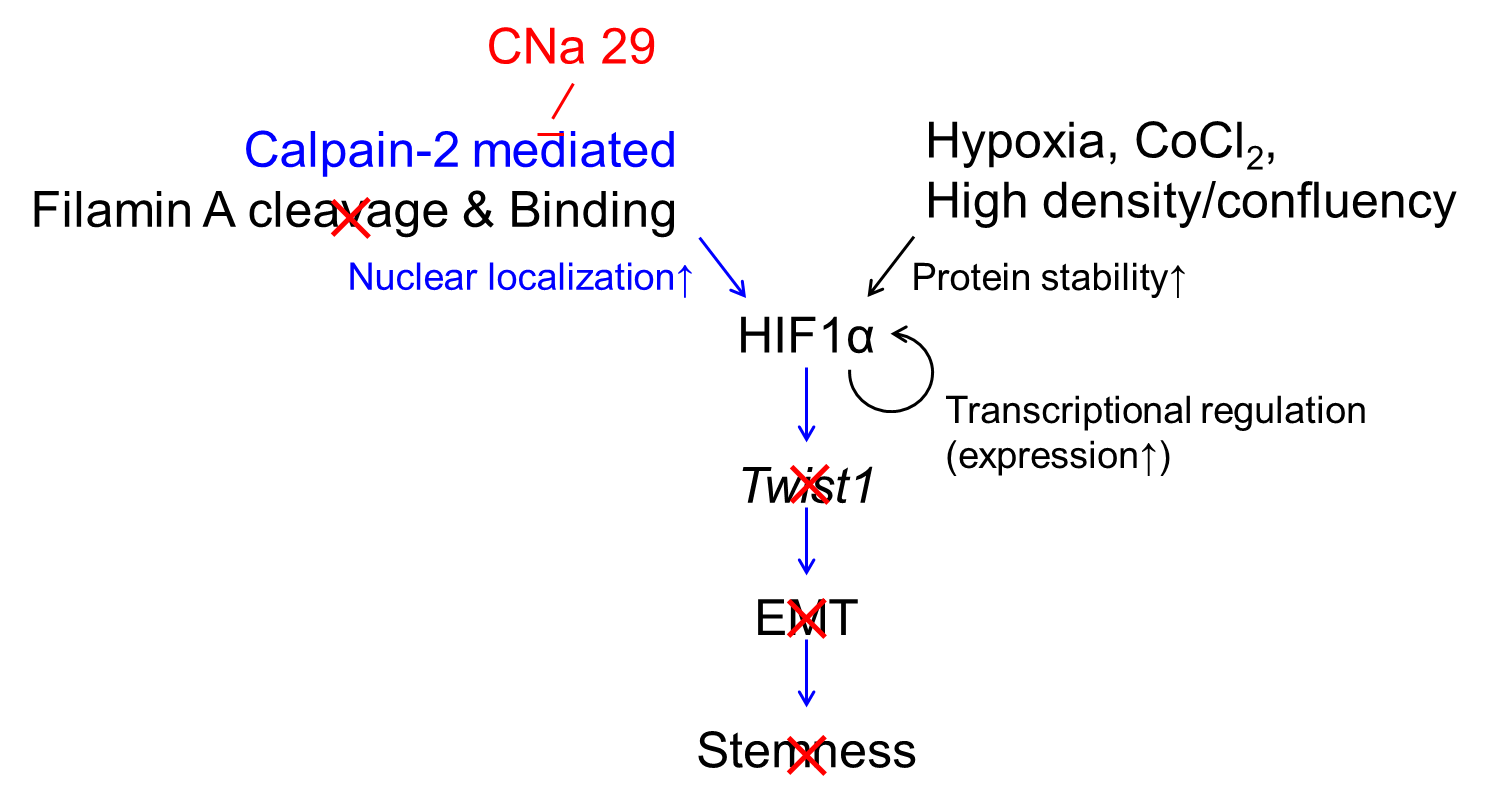


**Figure S13. Schematic diagram about the action of CNa 29.**


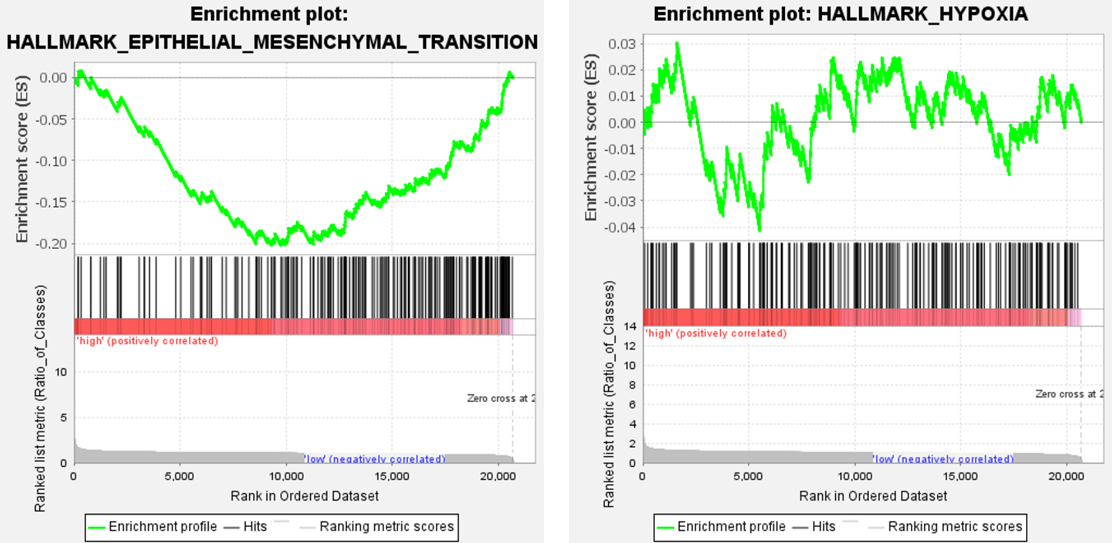


**Figure S14. Enrichment of EMT (left) and hypoxia (right) hallmark gene sets observed in *CAPN1* highly expressing TNBC patients.**

**Figure S15. Relative expression of FLNA in each subtype of breast cancer.**
